# Proinflammatory and cytotoxic CD38^+^HLA-DR^+^ effector memory CD8^+^ T cells are peripherally expanded in human cardiac allograft vasculopathy

**DOI:** 10.1016/j.ajt.2025.10.015

**Published:** 2025-10-24

**Authors:** Yuko Tada, Sujit S.A. Suthahar, Payel Roy, Vasantika Suryawanshi, Runpei Wu, Erpei Wang, Felix S. Nettersheim, Anusha Bellapu, Katarzyna Dobaczewska, Cheryl Kim, Florin Vaida, Gerald P. Morris, Klaus Ley, Paul J. Kim

**Affiliations:** 1 Division of Cardiovascular Medicine, University of California, San Diego, San Diego, California, USA; 2 La Jolla Institute for Immunology, La Jolla, California, USA; 3 Immunology Center for Georgia, Augusta University, Augusta, Georgia, USA; 4 Department of Cardiology, Faculty of Medicine and University Hospital Cologne, University of Cologne, Cologne, Germany; 5 Department of Family Medicine and Public Health, University of California, San Diego, San Diego, California, USA; 6 Department of Pathology, University of California, San Diego, San Diego, California, USA

**Keywords:** heart transplant, cardiac allograft vasculopathy, CD8^+^ T cells

## Abstract

Interferon gamma (IFNG) is thought to play a central role in the pathogenesis of cardiac allograft vasculopathy (CAV) in patients with heart transplant (HTx). However, peripheral lymphocytes participating in the IFNG axis remain largely unknown in human CAV. Using peripheral blood mononuclear cells from International Society for Heart and Lung Transplant grade 2 or 3 CAV (high-grade CAV) and normal patients with HTx, we performed high-dimensional analysis (high-grade CAV, n = 6; normal HTx, n = 12) with cellular indexing of transcriptomes and epitopes using sequencing and variability, diversity, and joining segment sequencing and validated the findings using flow cytometry in an independent cohort (high-grade CAV, n = 11; normal HTx, n = 12). Among the major immune cell populations, CD8^+^ T cells expressed IFNG most highly. Among the CD8^+^ T cell clusters, the CD38^+^HLA-DR^+^ CD8^+^ effector memory T cell cluster was significantly increased in CAV compared with normal HTx peripheral blood mononuclear cell samples. This cluster showed clonal expansion, increased IFNG signaling, and enhanced cytotoxicity with granzyme B and perforin 1 overexpression. CD38^+^HLA-DR^+^ CD8^+^ T cells also infiltrated the intima of explanted CAV coronary arteries. Thus, we concluded that circulating CD38^+^HLA-DR^+^ CD8^+^ effector memory T cells may contribute to the pathogenic IFNG axis in human CAV.

## 1. Introduction

Cardiac allograft vasculopathy (CAV) limits adult heart transplant (HTx) recipients’ longevity to a median survival of 12 years after HTx.^1^ However, the immune cells that are key to the pathogenesis of human CAV remain undetermined. Consequently, treatment of CAV in patients is limited using contemporary immunosuppressive regimens.^2^

CAV is considered to be mediated by chronic allogeneic immune responses, where interferon gamma (IFNG) plays a central role.^3^ Clinical studies have also shown that persistent production of donor-specific antibody (DSA) and recurrent episodes of antibody-mediated rejection (AMR) are associated with a greater risk of CAV.^4,5^ However, previous research has suggested that CAV is not solely mediated by conventional DSA-producing B cells.^6,7^ There is evidence from animal models that CD8^+^ T cells may contribute to the pathogenesis of allograft vasculopathy through the IFNG axis, independent of B cells.^8,9^ In studies that analyzed human CAV samples, coronary arteries from explanted hearts also showed prominent infiltration of CD4^+^ and CD8^+^ T cells with a helper type 1 immune response.^3,10,11^

Circulating lymphocytes participating in the IFNG-axis have been studied to a limited extent in human CAV. Activated circulating lymphocytes may contribute to the IFNG axis by secreting inflammatory cytokines and infiltrating into the cardiac allograft.^10,12^ Two previous studies have shown a positive correlation between CAV and circulating T cells expressing IFNG.^13,14^ In contrast, a recent study using single-cell RNA sequencing (RNA-seq) in peripheral immune cells did not show significant differential expression of IFNG in high-grade compared with patients with low-grade CAV.^15^

In this study, we sought to (1) identify circulating lymphocyte populations participating in the IFNG-axis in high-grade CAV using cellular indexing of transcriptomes and epitopes by sequencing (CITE-seq) and variability, diversity, and joining (VDJ) segment sequencing (VDJ-seq); (2) characterize the phenotype and clonality of key immune cell populations in patients with HTx; and (3) validate the findings using flow cytometry in an independent patient cohort.

## 2. Materials and Methods

### 2.1. Supporting Information

Additional information regarding materials and methods is available online in the Supplementary Materials. Reagents used in the experiments are listed in Supplementary Table 1. A full list of software packages and versions utilized for this study is detailed in Supplementary Table 2.

### 2.2. Study Design and Sample Collection

**Figure 1.**
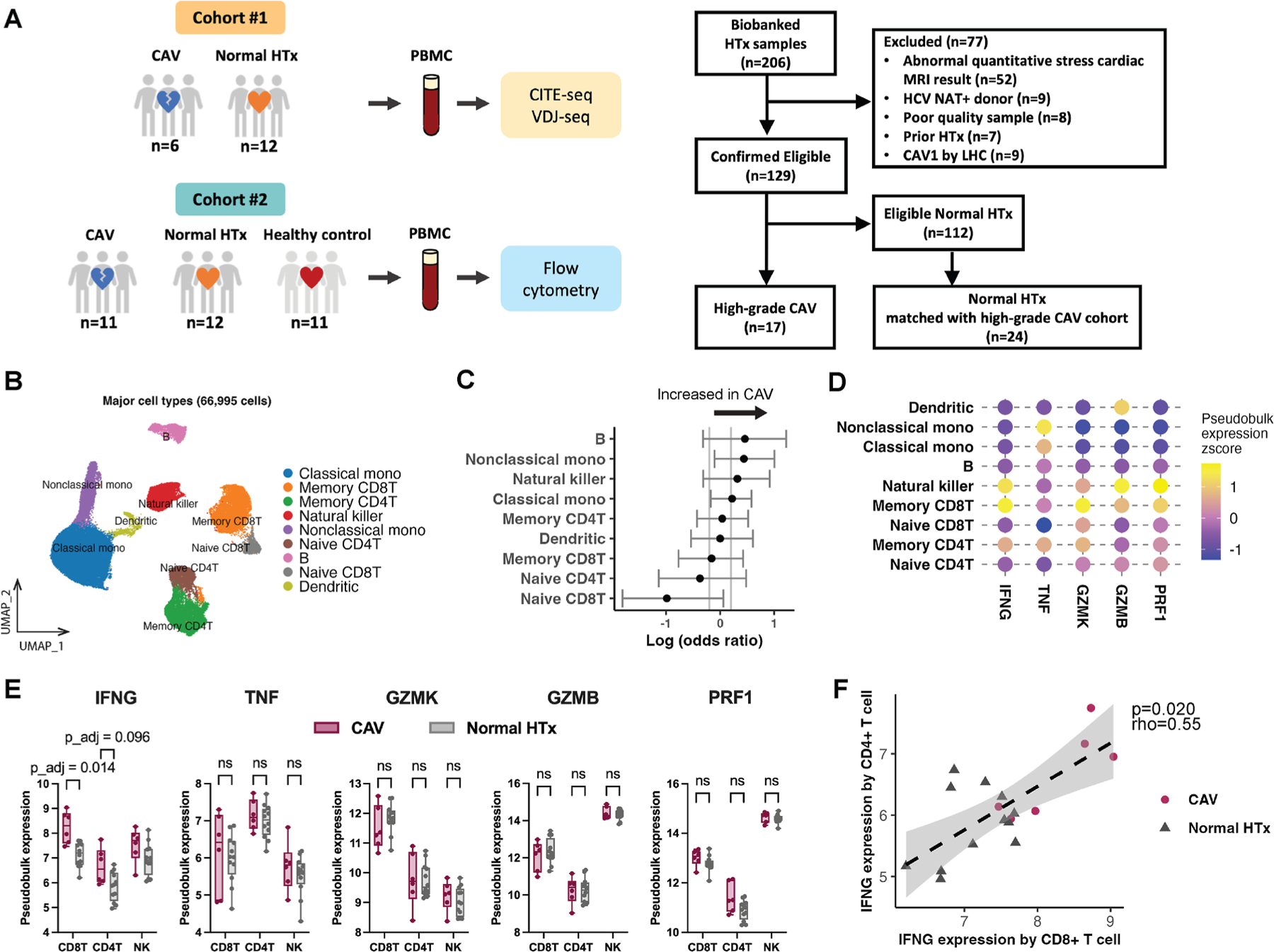
Major immune cell type analysis of CITE-seq shows the highest IFNG expression by circulating memory CD8^+^ T cells in patients with HTx. (A) Left, schematic representation of the study cohort and experimental design. PBMCs obtained from patients with high-grade CAV were divided into CITE-seq (cohort 1) and flow cytometry (cohort 2) cohorts. Right, the flow chart displays the selection criteria from biobanked HTx samples at UCSD. Patients with normal HTx were frequency matched with the high-grade CAV cohort based on age, sex, race, multiorgan transplant status, and time after HTx. The healthy control (HC) group without HTx consisted of young donors (<50 years old) recruited through the Normal Blood Donation Program at the La Jolla Institute for Immunology and older donors (≥50 years old) with a 0 coronary calcium score from UCSD. (B) The major circulating immune cell types obtained by unsupervised clustering are displayed on the UMAP embedding. (C) Log odds ratio estimates of differential major immune cell compositions are shown with 95% confidence intervals. No significant difference in peripheral major immune cell types was seen when comparing patients with high-grade CAV to patients with normal HTx. (D) Dot plot comparing average pseudobulk z-score expression of inflammatory and cytotoxic markers. Memory CD8^+^ T cells showed the highest IFNG expression among the major immune cell types. (E) Log-normalized pseudobulk expression values of inflammatory and cytotoxic markers by CD8^+^ T, CD4^+^ T, and NK cells are compared between high-grade CAV (n = 6) and normal HTx (n = 12) groups (Wilcoxon rank-sum test with *P* values adjusted using Bonferroni correction). IFNG expression was significantly increased in CD8^+^ peripheral T cells in patients with high-grade CAV compared with patients with normal HTx. (F) Pseudobulk IFNG expression in CD8^+^ and CD4^+^ T cells showed a significant positive correlation (Spearman correlation). CAV, cardiac allograft vasculopathy; CITE-seq, cellular indexing of transcriptomes and epitopes by sequencing; HC, healthy control; HCV, hepatitis C virus; HTx, heart transplant; IFNG, interferon gamma; GZMB, granzyme B; GZMK, granzyme K; LHC, left heart catheterization; NAT, nucleic acid test; PBMC, peripheral blood mononuclear cell; PRF1, perforin-1; TNF, tumor necrosis factor; UCSD, University of California, San Diego; UMAP, uniform manifold approximation and projection.

The overall study design is displayed in Figure 1A, and the patient recruitment strategy is shown in Supplementary Figure S1. Sample size was based on resource availability, and no statistical methods were used to predetermine sample size. Biobanked peripheral blood mononuclear cell (PBMC) samples were obtained between 2020 and 2023 from HTx recipients who were 18 years of age or older at the University of California, San Diego (UCSD). Exclusion criteria were HTx from hepatitis C nucleic acid test-positive donors, patients with a previous HTx, or poor-quality samples (eg, cell viability ≤ 80%, hemolysis, or excessive turbidity). Patients with HTx diagnosed with the International Society of Heart and Lung Transplant (ISHLT) grade 2 or 3 CAV were recruited in the high-grade CAV group,^16^ and all samples were collected within 3 months after CAV diagnosis. A subset of patients, who did not show ISHLT grade 2 or 3 CAV on previous coronary angiography, had a left ventricular ejection fraction (LVEF) > 50%, and exhibited no signs/symptoms of cardiac allograft dysfunction, were referred for a quantitative stress cardiac magnetic resonance imaging (MRI) scan to screen for microvascular dysfunction^17^ as part of a separate multicenter study.^18^ PBMC samples were collected at the time of the cardiac MRI scan. Patients with HTx with normal stress cardiac MRI scan results and without prior diagnosis of ISHLT grades 1 to 3 CAV were included in the normal HTx group. High-grade CAV and patients with normal HTx were frequency matched for age, sex, race, time after HTx, and multiorgan transplant status for CITE-seq and flow cytometry. The healthy control (HC) group without HTx consisted of young donors (<50 years old) recruited through the Normal Blood Donation Program at the La Jolla Institute for Immunology and older donors (≥50 years old) with a zero coronary calcium score from UCSD. The HC donors were screened for comorbidities, including hypertension, dyslipidemia, diabetes mellitus, and chronic viral infections (ie, hepatitis B virus, hepatitis C virus, and human immunodeficiency virus). This study was approved by the UCSD Office of Institutional Review Board Administration (No. 160 808), and all participants provided written informed consent. This study adheres to the principles of the Declaration of Helsinki and the ISHLT Statement on Transplant Ethics.

### 2.3. Sequencing

CITE-seq and VDJ-seq were performed using the BD Rhapsody single-cell analysis system.^19^ Cell surface epitopes were labeled with 56 antibody-derived tags (ADT) containing the AbSeq Immune discovery panel (30 antibodies) and 26 additional AbSeq antibodies (Supplementary Table 3). One high-grade CAV sample and 2 normal HTx samples were pooled using hashtagging and processed together to generate a total of 6 batches (Supplementary Table 4). Libraries of messenger RNA (mRNA), sample tag, AbSeq, and VDJ regions were prepared according to the manufacturer’s instructions. Polymerase chain reactions were performed for targeted mRNA amplification using the BD Human Immune Response Panel (398 genes) and a custom panel of ~100 genes (Supplementary Excel file S1). All samples were sequenced together using the NovaSeq 6000 S4 sequencer with 75 × 75 bp paired reads for mRNA, AbSeq, and sample tag libraries, and the SP sequencer with 75 × 225 bp paired reads for VDJ libraries.

### 2.4. Sequencing data analysis

#### 2.4.1. Clustering and differential expression analysis

CITE-seq data were analyzed using Seurat.^20^ ADT data were transformed to a centered log-ratio scale, and mRNA data were normalized to a log scale. Batch effects were corrected for based on the canonical correlation analysis (Supplementary Fig. S2A).^21^ For unsupervised clustering of major cell types, a k-nearest neighbors method and modularity optimization were performed using the Louvain algorithm (resolution = 0.2). For unsupervised clustering of CD8^+^ T, CD4^+^ T, B, and natural killer (NK) cells, weighted nearest neighbor (WNN) analysis was performed (resolution = 0.3).^20^ Remaining doublet clusters and unidentifiable small clusters (n < 50) were removed. Clusters were visualized using the uniform manifold approximation and projection (UMAP) dimensionality reduction. Reference-based cell annotations were performed using SingleR and Celldex.^22^ Logistic regression analysis for differential cell composition was performed using sccomp.^23^ Differential gene expression was analyzed using Find(All)Markers functions in Seurat to obtain log2 fold change expression and *P* values calculated from the MAST algorithm adjusted for the individual sample as a covariate. For pseudobulk gene expression analysis, aggregated raw count data were log-normalized by counts per million and corrected for batch effect using edgeR. Gene ontology for biological processes and gene set ordinal association analysis were performed using clusterProfiler and GOAT.^24,25^ The diffusion map was analyzed using destiny.^26^

#### 2.4.2. VDJ-seq data analysis

Clonality was evaluated using the paired CDR3 α and β chain amino acid sequences (CDR3αβ). Diversity of the CDR3αβ chains was standardized to adjust for different sample sizes by calculating the asymptotic diversity using bootstrapped rarefaction/extrapolation curves with iNEXT.^27^

### 2.5. Flow cytometry

Spectral flow cytometry data were acquired using the Cytek Aurora (Cytek Biosciences) and analyzed using FlowJo (BD Biosciences and FlowJo LLC).

### 2.6. Histologic immunofluorescence staining

Tissue samples obtained from hearts explanted for re-HTx due to ISHLT grade 3 CAV were used for immunostaining. Non-HTx control cardiac samples were obtained from healthy deceased donors through Lifesharing, San Diego, CA, USA. Two immunofluorescence panels targeting CD8/CD38/HLA-DR or CD4/CD38/HLA-DR proteins were used.

### 2.7. Statistical Analysis

The statistical tests performed are stated for each figure. Statistical analysis was performed using either R (R Core Team, 2024) or GraphPad Prism. Given the small sample sizes for each group, normality was not assessed, and nonparametric tests were utilized. Wherever multiple comparisons were performed, *P* values were adjusted using Bonferroni correction or the Benjamini-Hochberg procedure. Adjusted or unadjusted *P* values were designated as *P*_adj or *P*, respectively. *P*_adj or *P* < .05 were considered significant.

## 3. Results

### 3.1. Clinical characteristics of the study cohort

For this study, we analyzed PBMC samples from the CITE-seq (cohort 1; n = 18; Fig. 1A; Table 1) and flow cytometry cohorts (cohort 2; n = 34; Table 2). In both cohorts compared with the normal HTx group, patients with high-grade CAV showed a significantly lower LVEF and a nonsignificant increase in prevalence for history of DSA positivity and prednisone and β-blocker use at the time of sample collection. There was also a trend in higher median time from transplant in high-grade CAV compared with patients with normal HTx for cohort 1. Four patients with high-grade CAV died, and 6 patients with high-grade CAV underwent re-HTx during the follow-up period (up until December 2024), whereas no patients with normal HTx experienced death or re-HTx.

### 3.2. Peripheral CD8^+^ T, CD4^+^ T, and NK cells expressed higher IFNG compared with other major immune cell types in patients with HTx

We first analyzed PBMC samples from cohort 1 obtained from patients with normal HTx (n = 12) and patients with high-grade CAV (n = 6) using CITE-seq and VDJ-seq. Overall, 69 106 cells passed quality control filters, and unsupervised clustering was subsequently performed using a final set of 18 ADT markers to obtain major blood immune cell lineages (Fig. 1B, Supplementary Figs. S2B and S3, and Excel file S2–3). The reference-based cell type annotations using RNA expression also closely approximated the major immune cell cluster assignments (Supplementary Fig. S2C). We found no significant difference in proportions of major cell types between high-grade CAV and normal HTx (Fig. 1C).

Memory CD8^+^ T cells expressed IFNG most highly among the major immune cell types, followed by NK and memory CD4^+^ T cells (Fig. 1D). Pseudobulk IFNG expression showed a significant increase in CD8^+^ and an increased trend in CD4^+^ T cells in patients with high-grade CAV compared with patients with normal HTx (Fig. 1E). A significant positive correlation of IFNG expression by CD8^+^ and CD4^+^ T cells was also demonstrated (Fig. 1F).

### 3.3. Circulating CD38^+^HLA-DR^+^ CD8^+^ effector memory T cells were significantly increased in patients with high-grade CAV compared with normal HTx

Having learned the potential contribution to the IFNG axis by CD4^+^ T, CD8^+^ T, and NK cells, we wished to further analyze these lymphoid lineage clusters in addition to B cells between the high-grade CAV and normal HTx groups. Subclusters were obtained by unsupervised WNN analysis and called based on the expression of surface markers and genes (Supplementary Excel files S2 and S3).

To subcluster CD8^+^ T cells, naive (1761 cells) and memory (10 619 cells) CD8^+^ T cell clusters were combined. After removal of double positive (CD4^+^CD8^+^) and double negative (CD4^−^CD8^−^) clusters (Supplementary Fig. S4A), the remaining CD8^+^ T cells were analyzed to identify 7 CD8^+^ T cell subclusters (Supplementary Figs. 2A and S4): naive (C3); mucosal-associated invariant T (MAIT; C6); 3 effector memory T cell (Tem)—Tem (C2), CD38^+^HLA-DR^+^ Tem (C4), and CD25^+^ Tem (C7); and 2 effector memory re-expressing CD45RA T cell (Temra) clusters—Temra (C1) and CD56^+^ Temra (C5). Differential compositional analysis showed significantly increased cells in the CD38^+^HLA-DR^+^ CD8^+^ Tem cluster (log odds ratio = 1.43; FDR < 0.025) in patients with high-grade CAV compared with patients with normal HTx.

**Figure 2.**
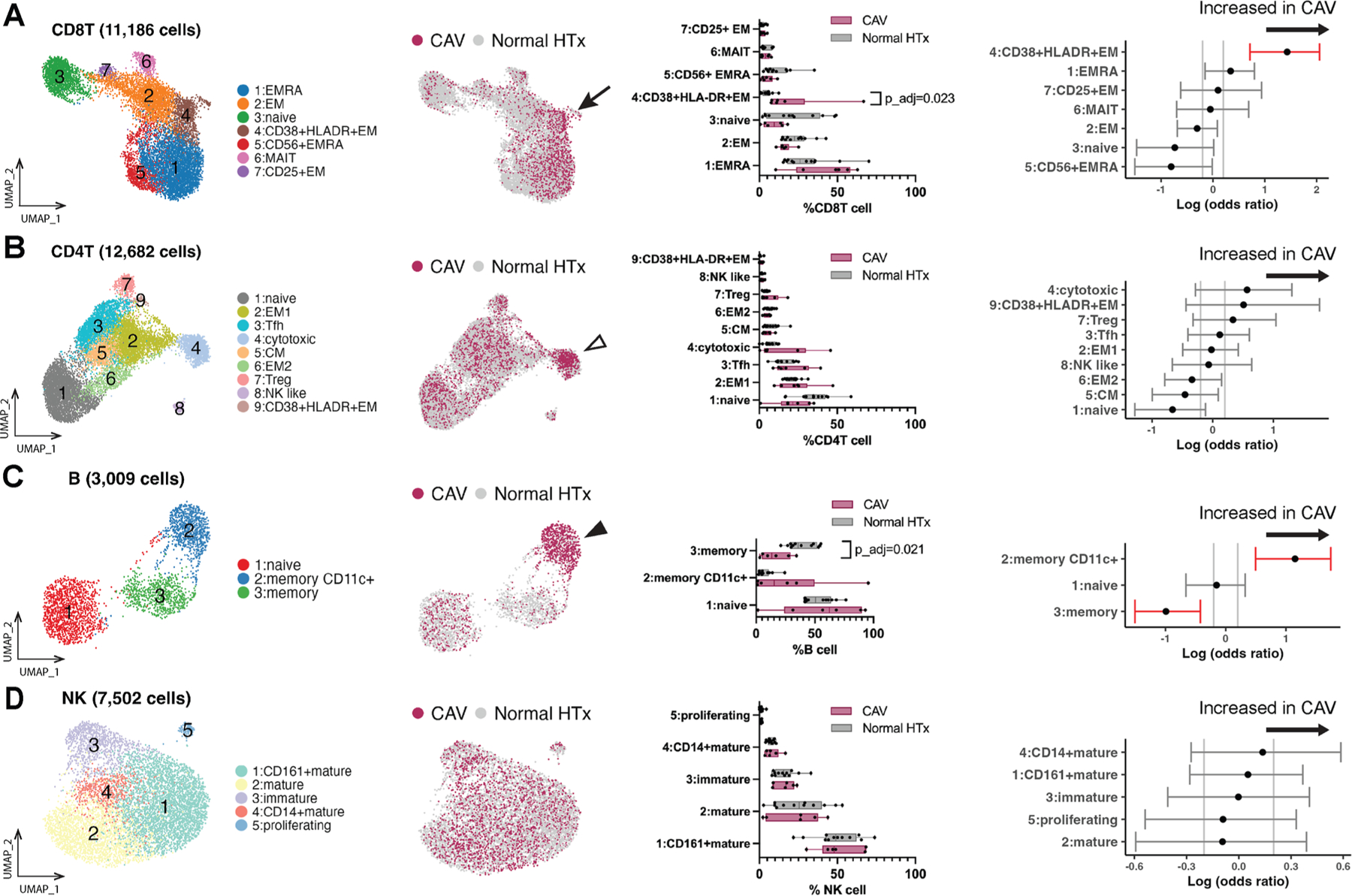
Analysis of peripheral CD8^+^ T cell clusters using CITE-seq reveals expansion of CD38^+^HLA-DR^+^ CD8^+^ Tem cells in patients with high-grade CAV. (A-D). Circulating CD8^+^ T (A), CD4^+^ T (B), B (C), and NK (D) cell clusters were identified across all PBMC samples in the CITE-seq cohort (n = 18) using unsupervised WNN analysis and displayed on the UMAP embeddings (left). Cells from the high-grade CAV vs normal HTx groups are annotated on the UMAP embeddings (second from the left). There was an increased distribution of immune cells from patients with high-grade CAV displayed in the CD38^+^HLA-DR^+^ CD8^+^ Tem (C4; black arrow), cytotoxic CD4^+^ T (C4: white arrowhead with black outline), and CD11c^+^ memory B cell clusters (C2: black arrowhead). Proportions of cells in each cluster (% of total CD8^+^ T, CD4^+^ T, B, and NK) were compared between the high-grade CAV and normal HTx groups (second from the right; Wilcoxon rank-sum test with FDR-adjusted *P* values using the Benjamini-Hochberg procedure). The CD38^+^HLA-DR^+^ CD8^+^ Tem cluster was significantly increased in patients with CAV (*P*_adj = .023). However, the increased proportion in the cytotoxic CD4^+^ T (*P*_adj = .981) or CD11c^+^ memory B (*P*_adj = .750) cell clusters was not found to be significantly different between patients with high-grade CAV and patients with normal HTx. Memory B cells were significantly decreased in patients with high-grade CAV vs patients with normal HTx (*P*_adj = .021). Log odds ratio estimates of differential cell compositions, displayed in the rightmost plots with 95% confidence intervals, demonstrated a significant increase in CD38^+^HLA-DR^+^ CD8^+^ Tem and CD11c^+^ memory B cells and a significant decrease in memory B cells in patients with high-grade CAV compared with patients with normal HTx. The red 95% confidence intervals indicate FDR < 0.025. CAV, cardiac allograft vasculopathy; CITE-seq, cellular indexing of transcriptomes and epitopes by sequencing; CM, central memory; EM, effector memory; EMRA, effector memory re-expressing CD45RA; HTx, heart transplant; MAIT, mucosal-associated invariant T; NK, natural killer; PMBC, peripheral blood mononuclear cell; Tem, effector memory T cell; Tfh, follicular helper T cell; Treg, regulatory T cell; UMAP, uniform manifold approximation and projection; WNN, weighted nearest neighbor.

To subcluster CD4^+^ T cells, naive (3895 cells) and memory (8816 cells) CD4^+^ T cells were combined. Nine CD4^+^ T cell subclusters were identified: naive (C1); 3 effector memory (C2, C6, and C9); circulating follicular helper (C3); cytotoxic (C4); central memory (C5); regulatory (C7); and NK-like (C8) clusters (Fig. 2B and Supplementary Fig. S5). No CD4^+^ T cell subclusters were found to be significantly different between patients with high-grade CAV and patients with normal HTx.

For B cells (3009 cells; Fig. 2C and Supplementary Fig. S6) and NK cells (7502 cells; Fig. 2D and Supplementary Fig. S7), 3 and 5 subclusters were identified, respectively. Differential compositional analysis showed significantly decreased memory B cells and significantly increased CD11c^+^ memory B cells in patients with high-grade CAV compared with patients with normal HTx. There was no significant compositional change in NK cells between the high-grade CAV and normal HTx groups.

### 3.4. CD38^+^HLA-DR^+^ CD8^+^ Tem cells showed high expression of proinflammatory and cytotoxicity markers

**Figure 3.**
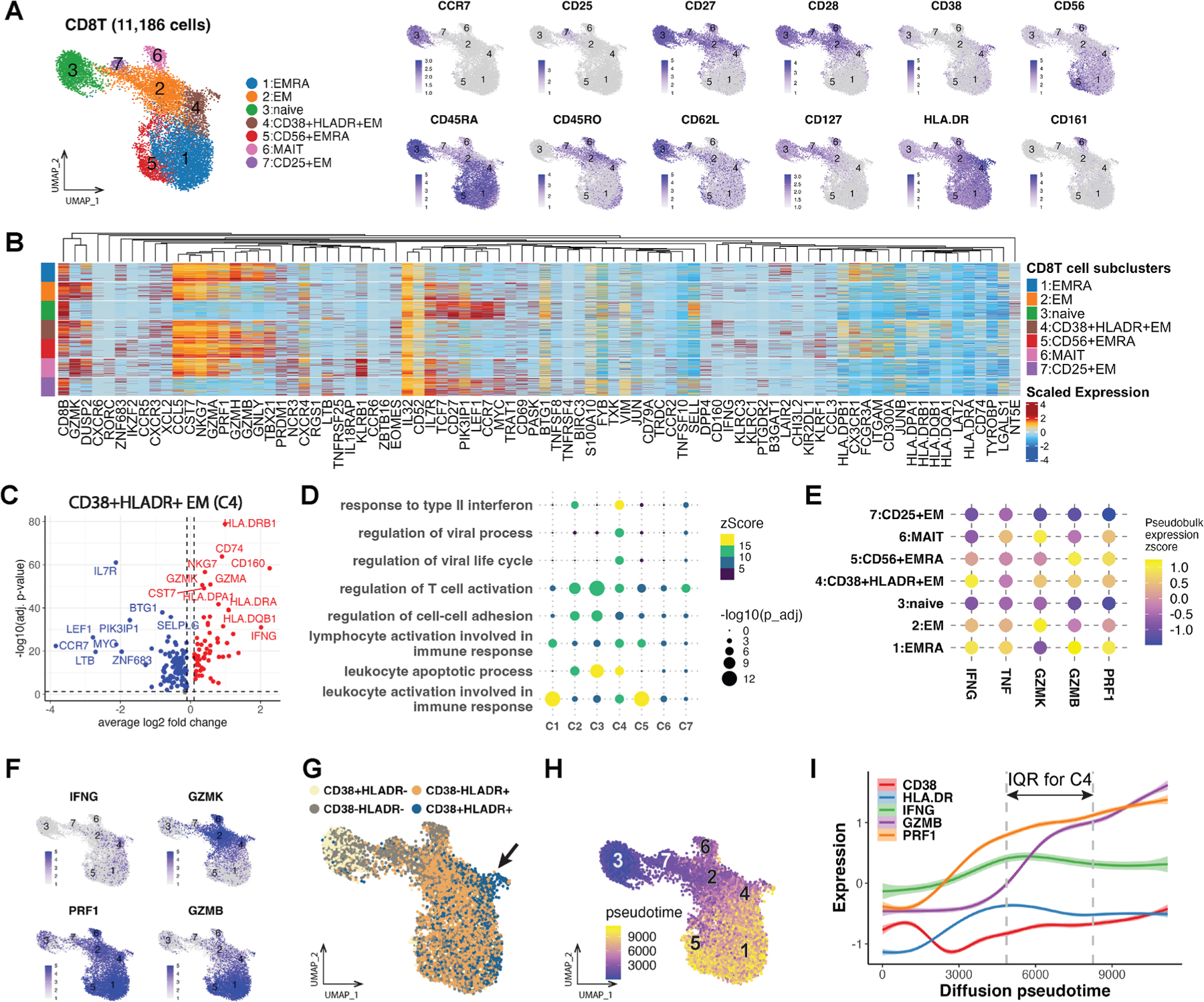
Transcriptomic analysis using CITE-seq shows upregulation of inflammatory and cytotoxic genes in circulating CD38^+^HLA-DR^+^ CD8^+^ Tem cells. (A) Feature plots of representative surface proteins for CD8^+^ T cell subclusters obtained from CITE-seq data using unsupervised WNN. (B) Heatmap of differentially expressed genes at the cell level among the CD8^+^ T cell subclusters. The heatmap was created using the ComplexHeatmap package in R. Cells were down-sampled to equal numbers across the subclusters. The color scale represents the scaled normalized expression values. (C) The volcano plot shows the differentially expressed genes in the CD38^+^HLA-DR^+^ CD8^+^ Tem cluster (C4). *P* values were adjusted by including the individual PBMC sample as a covariate and using the Benjamini-Hochberg procedure for FDR-adjusted *P* values. (D) Dot plot comparing enriched gene ontology of biological processes across CD8^+^ T cell subclusters. Activation of the type II interferon response is demonstrated in C4. The color scales indicate enrichment’s z-score. *P* values were adjusted using the Benjamini-Hochberg procedure for FDR-adjusted *P* values. (E) Dot plot compares pseudobulk expression of inflammatory and cytotoxic genes across CD8^+^ T cell subclusters and shows increased IFNG, GZMB, and PRF1 expression in C4 compared with other CD8^+^ Tem subclusters. (F) Feature plots of IFNG, GZMK, PRF1, and GZMB expression across CD8^+^ T cell subclusters. (G) CD38 and HLA-DR expression groups are annotated on the UMAP embedding of CD8^+^ subclusters. C4 predominantly consists of CD38^+^HLA-DR^+^ cells (black arrow). (H) The diffusion pseudotime, determined as the ranks of cells ordered along the diffusion trajectories, is annotated on the UMAP embedding displaying the CD8^+^ subclusters. C4 showed diffusion pseudotimes intermediate to other CD8^+^ T cell subclusters. (I) Trend lines of scaled expression (y-axis) for CD38, HLA-DR, IFNG, GZMB, and PRF1 were plotted along the CD8^+^ T cell diffusion pseudotime (x-axis). The vertical dashed gray lines represent the first and third quartiles (ie, IQR) of diffusion pseudotimes for C4. CD38 expression was initially high in naive CD8^+^ T cells and subsequently declined in memory cells (C2, C6, and C7) before rising in C4. This rise in CD38 expression in C4 was also associated with increased HLA-DR, IFNG, GZMB, and PRF1 expression compared with CD8^+^ T cell subclusters with earlier diffusion pseudotimes (C2, C3, C6, and C7). CITE-seq, cellular indexing of transcriptomes and epitopes by sequencing; EM, effector memory; EMRA, effector memory re-expressing CD45RA; GZMK, granzyme K; GZMB, granzyme B; IFNG, interferon-γ; IQR, interquartile range; MAIT, mucosal-associated invariant T; PMBC, peripheral blood mononuclear cell; PRF1, perforin-1; Tem, effector memory T cell; WNN, weighted nearest neighbor.

As CD38^+^HLA-DR^+^ CD8^+^ Tem cells (C4) were significantly increased in patients with high-grade CAV, we further explored this subcluster by gene expression analyses. Surface protein expression of CD38 and HLA-DR, and downregulation of CD28 by this subcluster compared with other CD8^+^ memory subclusters, were suggestive of its activated status (Fig. 3A and Supplementary Fig. S4D). The CD38^+^HLA-DR^+^ CD8^+^ Tem subcluster overexpressed genes associated with major histocompatibility complex class II (HLA.DRB1, HLA.DRA, HLA. DQB1, and CD74) and type II IFN-response (IFNG) as well as immune checkpoint regulation (CD160; Fig. 3B, C, and Supplementary Fig. S8). Gene ontology and gene set ordinal association analyses also demonstrated the upregulated type II IFN response by the CD38^+^HLA-DR^+^ CD8^+^ Tem cluster (Fig. 3D and Supplementary Fig. S8B). The CD38^+^HLA-DR^+^ CD8^+^ Tem cluster expressed IFNG at the highest level among all T and NK cell subclusters (Fig. 3E and Supplementary Fig. S8C). The CD38^+^HLA-DR^+^ Tem subcluster also expressed higher levels of the cytotoxic enzymes, granzyme B (GZMB) and a lower expression of granzyme K compared with other CD8^+^ memory T cell subclusters (Fig. 3F).

Next, we compared CD38 and HLA-DR surface protein expression patterns among the CD8^+^ T cell subclusters by thresholding their expression (Supplementary Fig. S9). The naive and Tem clusters showed distinct CD38 and HLA-DR expression patterns (Fig. 3G). We then evaluated the expression patterns of CD38 and HLA-DR using diffusion pseudotime as an estimated distance from naive cells (Fig. 3H). CD38 and HLA-DR surface protein expression plotted against diffusion pseudotime showed that CD38 initially decreased in memory compared with naive CD8^+^ T cells. As diffusion pseudotime values increased in memory cells, HLA-DR expression increased before the subsequent rise in CD38 expression (Fig. 3I). This rise in CD38 expression was associated with increased IFNG, GZMB, and perforin-1 (PRF1) expression. IFNG and HLA-DR expression peaked in the CD38^+^HLA-DR^+^ CD8^+^ Tem subcluster before demonstrating a gradual decline in expression observed in the Temra clusters.

### 3.5. Circulating CD38^+^HLA-DR^+^ CD8^+^ Tem cells demonstrated clonal expansion

**Figure 4.**
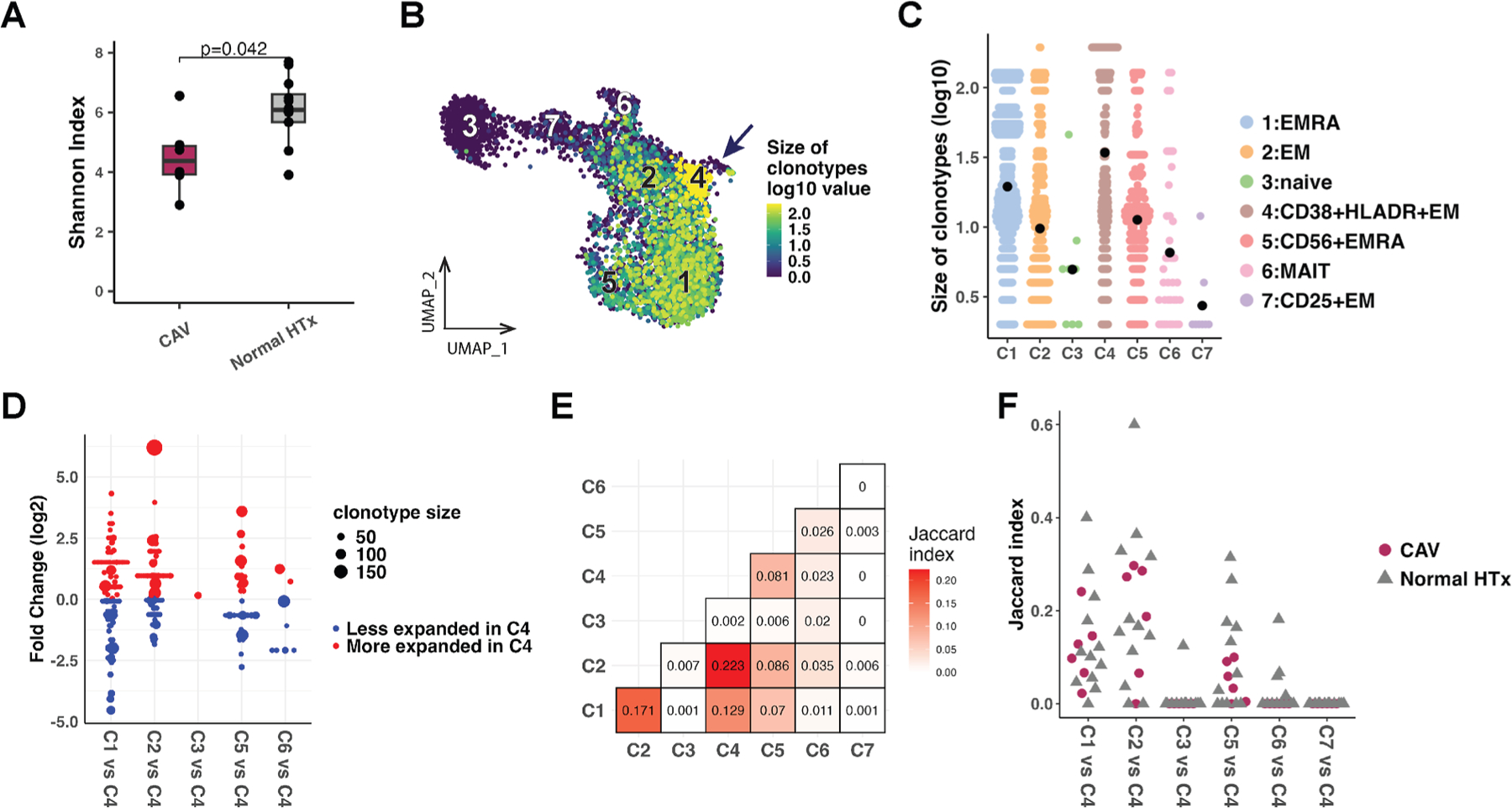
Circulating CD38^+^HLA-DR^+^ CD8^+^ Tem cells demonstrated clonal expansion and shared clonotypes with CD8^+^ T cell subclusters 1 and 2 using VDJ-seq. (A) The Shannon diversity index showed significantly decreased CD8^+^ TCR repertoire diversity in patients with high-grade CAV (n = 6) compared with patients with normal HTx (n = 12) (Wilcoxon rank-sum test). (B) The size of clonotypes (log10 scale) demonstrates the distribution of large clonotypes in the CD38^+^HLA-DR^+^ CD8^+^ Tem cell subcluster on the CD8^+^ UMAP embedding (C4, arrow). (C) The size of expanded clonotypes (log10 scale) is compared among the CD8^+^ T cell subclusters. The filled black circles represent the mean values. (D) Clonotypes expanded in C4 and shared with other CD8^+^ T cell subclusters are filtered, and similarities of the clonotypes are compared among the subclusters. Shared clonotypes are plotted across the relative proportion of cells in C4 vs non-C4 subclusters (log2 fold change). The size of each filled circle represents the size of each clonotype. Proportions of the cells were normalized by the size of the subclusters. The shared clonotypes with C4 were predominantly found in the Temra (C1) and Tem (C2) subclusters. (E) The heatmap shows the Jaccard index comparing the similarities of clonotypes between the subclusters. The values are the means of the Jaccard similarity index calculated for each sample (n = 18). (F) Similarity of clonotypes in C4 compared with other CD8^+^ T cell subclusters by the Jaccard index is displayed according to individual patient samples. Each filled circle or triangle represents a patient sample (n = 18). Clonotypes in C4 show higher similarity with those in C1 and C2 for patients with high-grade CAV and patients with normal HTx. CAV, cardiac allograft vasculopathy; EM, effector memory; EMRA, effector memory re-expressing CD45RA; HTx, heart transplant; MAIT, mucosal-associated invariant T; TCR, T cell receptor; Tem, effector memory T cell; Temra, effector memory re-expressing CD45RA T cell; UMAP, uniform manifold approximation and projection.

We then evaluated the expansion of the CDR3αβ clonotypes between the high-grade CAV and normal HTx groups and also among the CD8^+^ T cell subclusters (Supplementary Fig. S10A). VDJ data were obtained for 10 108 CD8^+^ T cells, 11 058 CD4^+^ T cells, and 2842 B cells. Patients with high-grade CAV demonstrated significantly reduced CD8^+^ T cell receptor diversity compared with normal HTx, consistent with increased clonal expansion of circulating CD8^+^ T cells (Fig. 4A). Of the total 2926 CD8^+^ T cell clonotypes, 11 (0.38%) were shared by no more than 2 patients, and no public clonotype across patients with high-grade CAV was identified. Patients with high-grade CAV also demonstrated significantly decreased diversity in CD4^+^ T cell receptors compared with normal HTx, but not in B cell receptors (Supplementary Fig. S10B–C).

The CD38^+^HLA-DR^+^ CD8^+^ Tem cell subcluster (C4) showed the largest average size of expanded clonotypes among the CD8^+^ T cell subclusters (Fig. 4B, C). When comparing overlapping T cell receptor repertoires, we found that clonotypes of C4 were shared by Temra (C1) and Tem (C2) subclusters (Fig. 4D). The mean Jaccard index showed that the clonotypes of C4 had the highest similarity with C2, which was followed by C1 (Fig. 4E, F).

### 3.6. Flow cytometry validated significantly increased circulating CD38^+^HLA-DR^+^ CD8^+^ Tem cells in patients with high-grade CAV compared with patients with normal HTx

**Figure 5.**
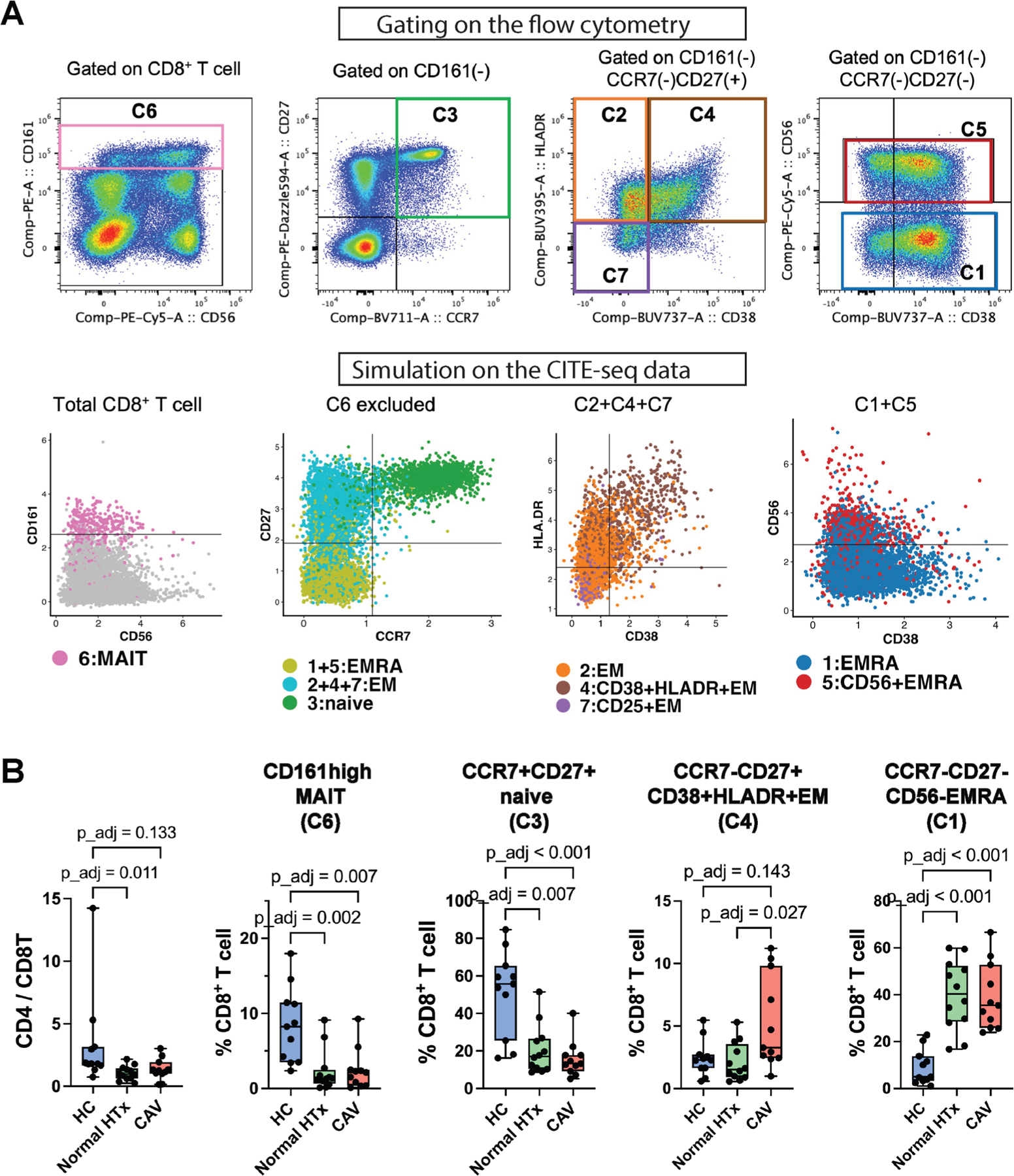
Flow cytometry validates CITE-seq results of an increased proportion of circulating CD38^+^HLA-DR^+^ CD8^+^ Tem cells in patients with high-grade CAV compared with patients with normal HTx. (A) Gating of CD8^+^ T cells for flow cytometry (top) was determined by simulations of the CITE-seq data (bottom) using cell surface markers to closely approximate the original subclusters. The top plots show representative gates on flow cytometry data. CD161^high^ MAIT CD8^+^ T (equivalent to C6) was gated first (left). CCR7 vs CD27 gating subsequently separated the non-C6 CD8^+^ T cells into naive, Tem, and Temra clusters (second from the left). CCR7^−^CD27^+^ Tem clusters were separated into C2, C4, and C7 by CD38 and HLA-DR expression (second from right). CCR7^−^CD27^−^Temra cells were then gated by CD56 to separate C1 and C5 (right). The bottom plots show the simulated gates on the CD8^+^ T cells using surface markers on the CITE-seq data. (B) Proportions of the gated CD8^+^ T cell subclusters (%CD8^+^ T cells) using flow cytometry are compared among high-grade CAV (n = 11), normal HTx (n = 12), and HC (n = 11) groups (Kruskal-Wallis test followed by the Dunn’s test, *P* values adjusted using Bonferroni correction). CD38^+^HLA-DR^+^ CD8^+^ Tem cells (C4) were significantly increased in the patients with high-grade CAV compared with normal HTx, validating our CITE-seq results. CAV, cardiac allograft vasculopathy; CCR7, C-C chemokine receptor type 7; CITE-seq, cellular indexing of transcriptomes and epitopes by sequencing; EM, effector memory; EMRA, effector memory re-expressing CD45RA; HC, healthy control; HTx, heart transplant; MAIT, mucosal-associated invariant T; Tem, effector memory T cell; Temra, effector memory re-expressing CD45RA T cell.

Next, we analyzed PBMC samples from cohort 2 obtained from patients with high-grade CAV (n = 11), patients with normal HTx (n = 12), and HC participants (n = 11; Table 2 and Supplementary Table 7, Supplementary Fig. S11, and Supplementary Excel file S4) using flow cytometry. We gated CD8^+^ T cells using cell surface markers CD161, CD27, CCR7, CD38, HLA-DR, and CD56 to reproduce the CITE-seq subclusters (Fig. 5A). Subclustering of CD8^+^ T cells using flow cytometry closely approximated subclusters obtained using CITE-seq using WNN (Supplementary Fig. S12A, B).

The CD4/CD8 T cell ratio was significantly decreased in patients with normal HTx compared with HC participants (Fig. 5B). The proportion of the CD38^+^HLA-DR^+^ CD8^+^ Tem cells was significantly increased in patients with high-grade CAV (median = 3.3%; interquartile range [IQR], 2.4%−9.8%; *P*_adj = .027) compared with patients with normal HTx (median = 1.5%; IQR, 0.9–3.6%), corroborating our initial CITE-seq findings (Fig. 5B). No significant association was found between the years post-HTx and CD38^+^HLA-DR^+^ CD8^+^ Tem cells in both cohorts 1 and 2 (Supplementary Fig. S12C, D).

Both patients with high-grade CAV and normal HTx demonstrated significantly decreased MAIT and naive CD8^+^ T cell populations and significantly increased Temra populations compared with HC participants.

### 3.7. Flow cytometry validated expression of both proinflammatory and cytotoxic markers by circulating CD38^+^HLA-DR^+^ CD8^+^ Tem cells

**Figure 6.**
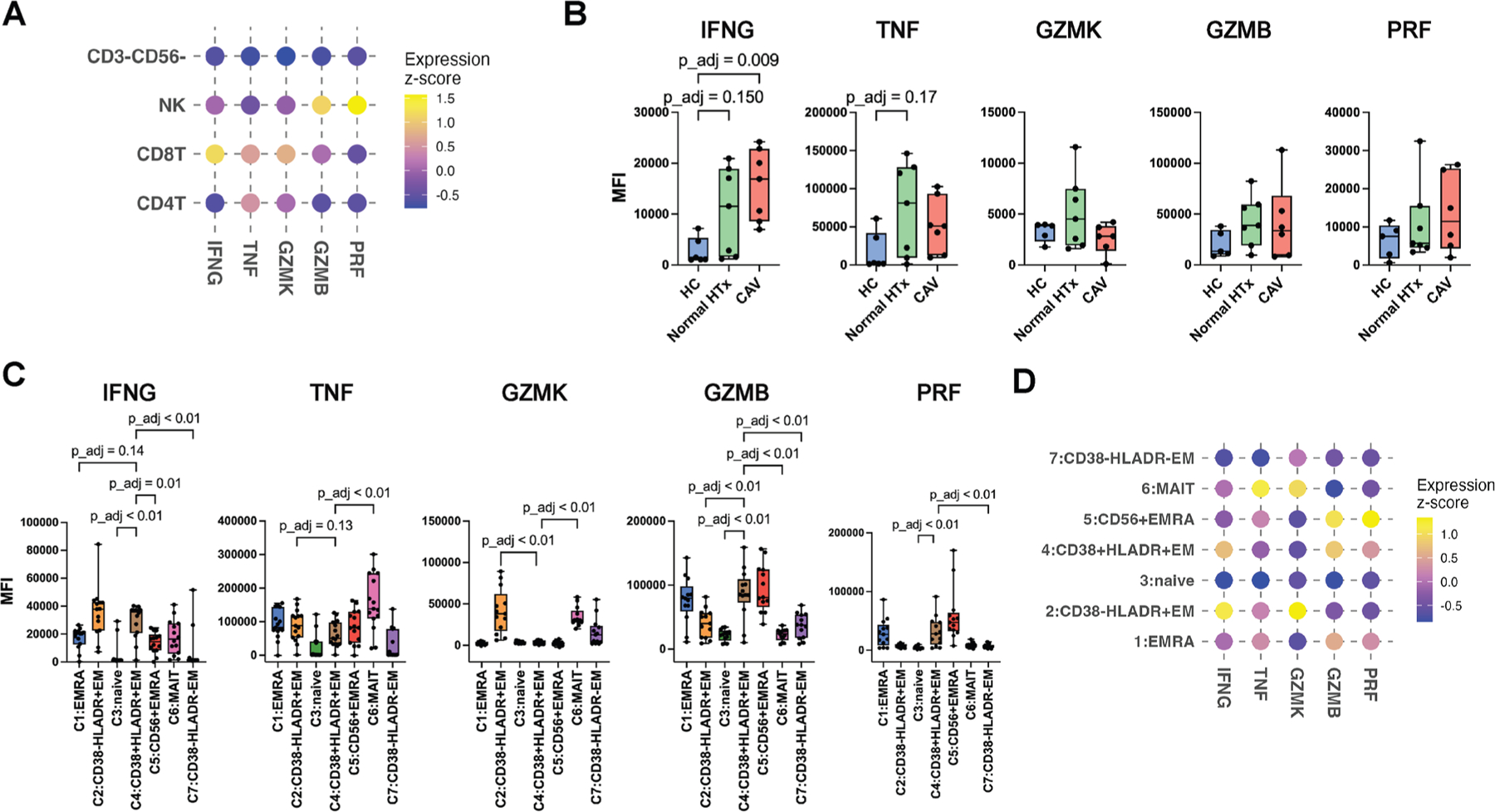
Intracellular staining validates CITE-seq results of increased IFNG, GZMB, and PRF expression in circulating CD38^+^HLA-DR^+^ CD8^+^ Tem cells. (A) Dot plot compares the average z-score for IFNG, TNF, GZMK, GZMB, and PRF expression among CD3^−^CD56^−^ (non-T, non-NK), NK, CD4^+^ T, and CD8^+^ T cells from both patients with high-grade CAV and normal HTx. IFNG was expressed at the highest level in CD8^+^ T cells. (B) Expression of the inflammatory and cytotoxic markers in CD8^+^ T cells was compared among the HC, normal HTx, and high-grade CAV groups. Patients with high-grade CAV showed significantly higher IFNG expression compared with HC participants. (C) Expression of intracellular markers is compared between C4 and the other CD8^+^ T cell subclusters, showing IFNG, GZMB, and PRF are highly expressed by C4. (D) A dot plot compares the scaled expression (z-score) of the intracellular markers among the CD8^+^ T cell subclusters. The color represents the average z-score. Sample numbers for IFNG and TNF: HC (n = 6), normal HTx (n = 7), and high-grade CAV (n = 7); for GZMK, GZMB, and PRF: HC (n = 5), normal HTx (n = 7), and high-grade CAV (n = 6). Statistical comparison was performed using the Kruskal-Wallis test followed by the post hoc Dunn’s test for B and the paired nonparametric Friedman test followed by the Dunn’s test for C (*P* values adjusted using Bonferroni correction for B and C). CAV, cardiac allograft vasculopathy; CITE-seq, cellular indexing of transcriptomes and epitopes by sequencing; EM, effector memory; EMRA, effector memory re-expressing CD45RA; GZMB, granzyme B; GZMK, granzyme K; HC, healthy control; HTx, heart transplant; IFNG, interferon-γ; MAIT, mucosal-associated invariant T; MFI, median fluorescence intensity; NK, natural killer; PRF, perforin; Tem, effector memory T cell; Temra, effector memory re-expressing CD45RA T cell; TNF, tumor necrosis factor.

Expression of inflammatory markers was compared by the median fluorescence intensity using flow cytometry with intracellular staining (Supplementary Figs. S13 and S14). In patients with HTx, we found the highest IFNG expression in CD8^+^ T cells and the highest expression of GZMB and PRF by NK cells (Fig. 6A), consistent with our CITE-seq findings.

Patients with high-grade CAV expressed significantly higher IFNG compared with HC participants in circulating CD8^+^ T cells (Fig. 6B). Circulating CD4^+^ T cells, but not NK cells, showed significantly higher IFNG expression in patients with high-grade CAV compared with HC participants (Supplementary Fig. S14A, B). A significant positive correlation of IFNG expression by CD8^+^ and CD4^+^ T cells was also validated in cohort 2 (Supplementary Fig. S14C). CD38^+^HLA-DR^+^CD8^+^ Tem (C4) cells demonstrated high IFNG, GZMB, and PRF expression (Fig. 6C, D), validating our CITE-seq findings.

### 3.8. CD38^+^HLA-DR^+^ CD8^+^ T cells were found in the intima of coronary arteries from patients with ISHLT grade 3 CAV

**Figure 7.**
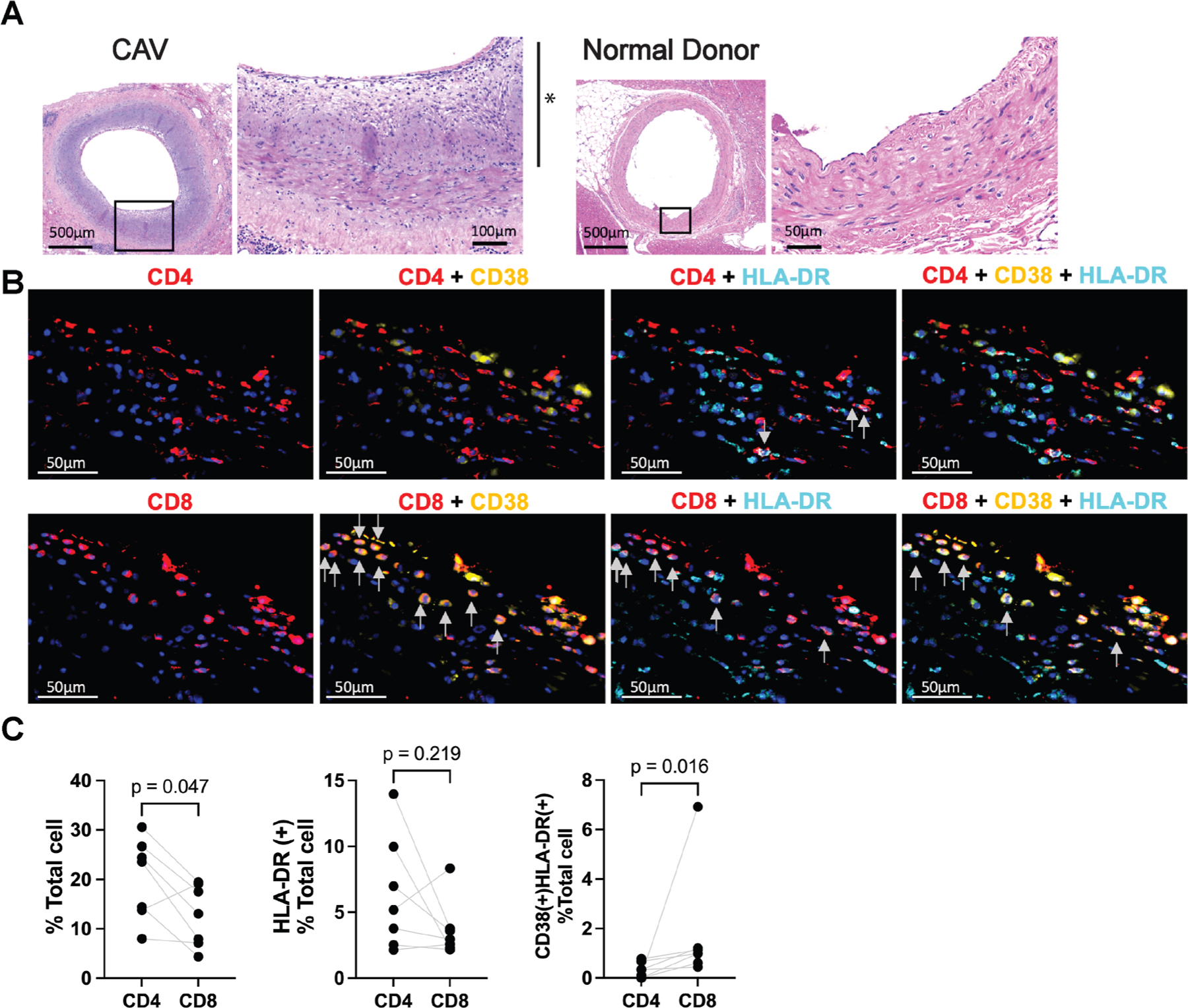
Immunofluorescence staining shows infiltration of CD38^+^HLA-DR^+^ CD8^+^ T cells in the intima of coronary arteries from patients with ISHLT grade 3 CAV. (A) Left, hematoxylin and eosin staining shows prominent intimal thickening of the coronary artery from a patient with ISHLT grade 3 CAV (indicated by *). Right, no intimal thickening is found in the representative normal donor coronary artery. The black boxes identify areas of interest shown at higher magnification to the right of the original images. (B) Representative immunofluorescence images compare CD4^+^ T (upper panel, red) and CD8^+^ T cells (lower panel, red) expressing CD38 (yellow) and/or HLA-DR (turquoise) in the intima of the coronary artery from a patient with ISHLT grade 3 CAV (nuclei are shown blue; arrows indicate positive cells). The equivalent intimal locations are shown for the CD4 and CD8 panels. CD38^+^HLA-DR^+^ CD8^+^ T cells are found in the intima of the allograft artery with ISHLT grade 3 CAV. (C) Spaghetti plots of individual pairs (quantified using immunofluorescence staining and pairs represented by connecting lines) compare proportions of CD4^+^ and CD8^+^ T (left), HLA-DR expressing CD4^+^ and CD8^+^ T (middle), and CD38 and HLA-DR expressing CD4^+^ and CD8^+^ T cells (right) that infiltrated the intima of coronary arteries from patients with ISHLT grade 3 CAV (n = 7). The y-axis represents the proportion of nucleated cells. CD38^+^HLA-DR^+^ cells were significantly more prevalent in CD8^+^ compared with CD4^+^ T cells. Statistical comparisons were performed using the paired Wilcoxon signed-rank test. CAV, cardiac allograft vasculopathy; ISHLT, International Society for Heart and Lung Transplant; HLA, human leukocyte antigen.

Lastly, we evaluated potential infiltration of CD38^+^HLA-DR^+^ CD8^+^ T cells in ISHLT grade 3 CAV (n = 7) and donor non-HTx myocardial tissue (n = 4). Prominent intima-media thickening with infiltration of CD4^+^ and CD8^+^ T cells was observed only in the CAV coronary arteries (Fig. 7A). CD38^+^HLA-DR^+^ CD8^+^ T cells were also identified in the intima of the coronary arteries for CAV samples (Fig. 7B). Although HLA-DR^+^ CD4^+^ T cells were found in the intima of patients with CAV, CD38^+^HLA-DR^+^ cells were significantly more prevalent in CD8^+^ T cells (Fig. 7C).

## 4. Discussion

This is the first study to fully investigate circulating lymphocytes contributing to the IFNG-axis in human CAV using CITE-seq and VDJ-seq with validation using flow cytometry in an independent patient cohort. We demonstrated activation of peripheral CD8^+^ T cells in patients with CAV with an increased proportion of the CD38^+^HLA-DR^+^ CD8^+^ Tem population compared with patients with normal HTx. CD38^+^HLA-DR^+^ CD8^+^ Tem cells were characterized by higher clonal expansion, activated IFNG pathway, and cytotoxicity with GZMB and PRF expression. Infiltration of the intima of CAV vessels by CD38^+^HLA-DR^+^ CD8^+^ T cells was also shown using immunostaining. Thus, our findings suggest circulating CD38^+^HLA-DR^+^ CD8^+^ Tem cells may contribute to the development of human CAV through the IFNG axis.

Although prior studies have provided evidence for the critical role of the IFNG axis in CAV,^28,29,^ the relative contribution of IFNG expression by CD4^+^ T, CD8^+^ T, and NK cells continues to be investigated.^30^ IFNG expressed by CD8^+^ T cells have been shown to mediate CAV in murine models by inducing smooth muscle cell apoptosis, and potentially initiating the vascular remodeling characterized by CAV.^8,9,31^ CD38^+^HLA-DR^+^ CD8^+^ T cells have previously been reported in other proinflammatory disease states, including atherosclerosis, HIV, and hepatitis C.^32–34^ Thus, our study findings newly establish the link between circulating CD38^+^HLA-DR^+^ CD8^+^ Tem cells and human CAV.

We also demonstrated the cytotoxic phenotype of circulating CD38^+^HLA-DR^+^ CD8^+^ Tem cells in human CAV. Previous studies have suggested that both IFNG and cytotoxic pathways are required to mediate allograft vasculopathy in calcineurin-treated mice.^8,35^ GZMB has also been shown to work together with PRF to induce endothelial cell apoptosis in the donor heart.^36,37^ We hypothesize that circulating CD38^+^HLA-DR^+^ CD8^+^ Tem cells infiltrate the intima of allograft arteries to cause intimal injury through proinflammatory and cytotoxic pathways, ultimately leading to CAV.

Recently, Amancherla et al^15^ performed single-cell RNA-seq in PBMCs but did not find a significant difference in CD8^+^ T cell subclusters in patients with high-grade CAV compared with patients with low-grade CAV.^15^ In addition, IFNG was not found to be differentially expressed among the circulating immune cell clusters. A possibility for the differences in the results of our study compared with Amancherla et al^15^ in part may be due to the differences in patient characteristics between the 2 study cohorts. Our patient cohort was older and male predominant, similar to the demographic characteristics of recent ISHLT registries.^38^ Additionally, we screened for microvascular CAV using quantitative stress cardiac MRI scans in our patients with a normal HTx. Importantly, our corroboration in an independent cohort using flow cytometry, as well as demonstration of CD38^+^HLA-DR^+^ CD8^+^ T cells using myocardial tissue immunostaining, provides confidence in the validity of our CITE-seq results.

Although we have demonstrated that CD38^+^HLA-DR^+^ CD8^+^ Tem cells are associated with human CAV, we could not determine the relationship of CD38^+^HLA-DR^+^ CD8^+^ Tem cells with DSA and AMR in this study. CAV likely represents a complex disease process where DSA and AMR contribute to varying degrees.^11,39–41^ To add to the complex nature of CAV, although we did not observe significant differences in peripheral CD4^+^ T cell subcluster proportions between patients with high-grade CAV and patients with normal HTx, memory CD4^+^ T cells, also demonstrating both inflammatory and cytotoxic profiles, have been shown by others to infiltrate the intima of allografts and mediate local inflammation in patients with CAV.^10,42,43^ In addition, we found a significant correlation between IFNG expression by peripheral CD4^+^ and CD8^+^ T cells that warrants further study. We also observed a significant depletion of memory B cells and an increase in exhausted memory B cells in high-grade CAV compared with patients with normal HTx. This finding could potentially be a consequence of long-lasting IFNG expression by CD4^+^ and CD8^+^ T cells.^44^ Thus, longitudinal studies are needed to further define the relationship between CD4^+^ T, CD8^+^ T, and B cells in human CAV.

As the mechanisms of CD38^+^HLA-DR^+^ CD8^+^ Tem cells contributing to CAV are further defined, identification of circulating CD38^+^HLA-DR^+^ CD8^+^ Tem cells could be a potential biomarker to predict high-grade CAV in patients with HTx. CD38^+^HLA-DR^+^ CD8^+^ Tem cells can be readily identified from PBMCs using flow cytometry in a clinical laboratory setting. Future studies should seek to determine whether CD38^+^HLA-DR^+^ CD8^+^ Tem cells predict the development of high-grade CAV.

### 4.1. Limitations

First, our study cohort was limited in the representation of patients with HTx with a history of AMR. As a result, this limited our study of the potential relationship between AMR and CAV. In addition, our cohort may not be fully representative of the heterogeneity of peripheral immune cell differences, including CD4^+^ T and NK cells, in high-grade CAV. Second, due to the limited sample size, we were unable to match samples for other potential confounders, including immunosuppression regimen at the time of sampling. However, we did not find a significant difference in mammalian target of rapamycin inhibitor use between patients with high-grade CAV and patients with normal HTx. We also excluded patients with CAV with a hepatitis C nucleic acid positive donor heart, or previous HTx, to avoid these potential confounders affecting peripheral immune cell response interpretation.^45,46^ Third, low-risk patients greater than 5 years post-HTx did not undergo coronary angiography if they were found to have a normal quantitative stress cardiac MRI scan. In support of this practice, a previous study has demonstrated the high diagnostic accuracy of using quantitative stress cardiac MRI scans for the detection of epicardial and/or microvascular CAV.^17^ Fourth, detailed gene set pathway analysis was limited due to our use of a restricted immune response gene panel for this study. Fifth, human cardiac allograft arteries from normal patients with HTx remain challenging to obtain and are a recognized limitation in this field of study.

## 5. Conclusion

We concluded that PBMCs from patients with high-grade CAV demonstrated significantly expanded CD38^+^HLA-DR^+^ effector memory CD8^+^ T cells with increased IFNG signaling and cytotoxicity compared with patients with normal HTx. Detection of circulating CD38^+^HLA-DR^+^ effector memory CD8^+^ T cells may be a useful biomarker for high-grade CAV.

## Supplementary Material

supp5

supp4

supp3

supp2

supp1

Appendix A. Supplementary data

Supplementary data to this article can be found online at https://doi.org/10.1016/j.ajt.2025.10.015.

## Figures and Tables

**Table 1:** Patient description for CITE-seq (cohort 1).

Characteristic	Normal HTx (n = 12)^a^	High-grade CAV (n = 6)^a^	*P*-value^b^
BMI (kg/m^2^)	27.3 (23.3–33.0)	26.8 (22.7–36.0)	.874
Age (y)	61.0 (54.0–67.2)	54.5 (39.2–66.5)	.599
Female sex	3 (25.0%)	1 (16.7%)	> .9
Time post-HTx (y)	5.5 (3.0–10.6)	16.2 (6.0–24.5)	0.119
CAV grade 0/1	12 (100%)	0 (0%)	< .001
2	0 (0%)	2 (33.3%)	
3	0 (0%)	4 (66.7%)	
Dual organ Tx (%)	2 (16.7%)	1 (16.7%)	> .9
HLA-A mismatches	2.0 (1.0–2.0)	2.0 (1.8–2.0)	.439
HLA-B mismatches	2.0 (2.0–2.0)	2.0 (1.8–2.0)	> .9
HLA-DR mismatches	1.0 (1.0–2.0)	1.0 (0.8–2.0)	.705
History of DSA	3 (27.3%)^c^	3 (75.0%)^d^	.235
History of ACR	0 (0%)	2 (50.0%)^d^	.050
History of AMR	1 (8.3%)	0 (0%)^d^	> .9
History of CMV viremia	4 (33.3%)	2 (50.0%)^d^	.604
LVEF (%)	65.5 (60.0–76.5)	54.5 (43.2–62.5)	.026
eGFR (mL/min/1.73m^2^)	62.0 (57.0–73.0)	51.5 (24.2–68.8)	.108
Diabetes	6 (50.0%)	3 (50.0%)	> .9
Hypertension	10 (83.3%)	5 (83.3%)	> .9
Calcineurin inhibitor use	12 (100%)	6 (100%)	> .9
Mycophenolate mofetil use	4 (33.3%)	2 (33.3%)	> .9
Prednisone use	1 (8.3%)	3 (50.0%)	.083
mTOR inhibitor use	9 (75.0%)	4 (66.7%)	> .9
Statin use	12 (100%)	5 (83.3%)	.333
ACEI or ARB use	4 (33.3%)	0 (0%)	.245
β-blocker use	0 (0%)	2 (33.3%)	.098
Outcomes			
Death	0 (0%)	2 (33.3%)	.098
Re-HTx	0 (0%)	4 (66.7%)	.005

ACEI, angiotensin-converting enzyme inhibitor; ACR, acute cellular rejection; AMR, antibody-mediated rejection; ARB, angiotensin receptor blocker; BMI, body mass index; CAV, cardiac allograft vasculopathy; CITE-seq, cellular indexing of transcriptomes and epitopes by sequencing; CMV, cytomegalovirus; DSA, donor-specific antibody; eGFR, estimated glomerular filtration rate; HLA, human leukocyte antigen; HTx, heart transplant; IQR, interquartile range; LVEF, left ventricular ejection fraction; mTOR, mammalian target of rapamycin; Tx, transplant.

a Median (IQR); n (%).

b Wilcoxon rank-sum test; Fisher exact test.

c Data missing for 1 patient.

d Data missing for 2 patients.

**Table 2:** Patient description for flow cytometry (cohort 2).

Characteristic	Healthy control (n = 11)^a^	Normal HTx (n = 12)^a^	High-grade CAV (n = 11)^a^	*P*-value^b^
BMI (kg/m^2^)	-	25.6 (22.4–31.0)	22.6 (19.7–25.4)	.138
Age (y)	46.0 (38.0–62.0)	62.0 (41.0–68.5)	56.0 (45.0–70.0)	.297
Female sex	4 (36.4%)	3 (25.0%)	1 (9.1%)	.368
Time post-HTx (y)	-	6.2 (1.6–8.0)	4.4 (2.2–5.9)	.515
CAV grade 0/1	-	12 (100%)	0 (0%)	< .001
2	-	0 (0%)	5 (45.5%)	
3	-	0 (0%)	6 (54.5%)	
Dual organ Tx (%)	3	1 (8.3%)	1 (9.1%)	> .9
History of DSA	-	3 (25.0%)	6 (54.6%)	.214
History of ACR	-	2 (16.7%)	4 (36.4%)	.371
History of AMR	-	1 (8.3%)	3 (27.3%)	.317
History of CMV viremia	-	4 (33.3%)	5 (45.4%)	.680
LVEF (%)	-	61.0 (59.0–64.8)	57.0 (54.0–61.0)	.033
eGFR (mL/min/1.73m^2^)	-	65.0 (55.5–82.8)	57.0 (21.0–85.0)	.324
Diabetes	-	6 (50.0%)	6 (54.6%)	> .9
Hypertension	-	9 (75.0%)	8 (72.7%)	> .9
Calcineurin inhibitor use	-	12 (100%)	11 (100%)	> .9
Mycophenolate mofetil use	-	1 (8.3%)	3 (27.3%)	.317
Prednisone use	-	2 (16.7%)	4 (36.4%)	.371
mTOR inhibitor use	-	10 (83.3%)	8 (72.7%)	.640
Statin use	-	11 (91.7%)	10 (90.9%)	> .9
ACEI or ARB use	-	6 (50.0%)	2 (18.2%)	.193
β-blocker use	-	0 (0%)	3 (27.3%)	.093
Outcomes				
Death	-	0 (0%)	2 (18.2%)	.217
Re-HTx	-	0 (0%)	2 (18.2%)	.217

ACEI, angiotensin-converting enzyme inhibitor; ACR, acute cellular rejection; AMR, antibody-mediated rejection; ARB, angiotensin receptor blocker; BMI, body mass index; CAV, cardiac allograft vasculopathy; CMV, cytomegalovirus; DSA, donor-specific antibody; eGFR, estimated glomerular filtration rate; HTx, heart transplant; IQR, interquartile range; LVEF, left ventricular ejection fraction; mTOR, mammalian target of rapamycin; Tx, transplant.

a Median (IQR); n (%).

b Wilcoxon rank-sum test; Fisher exact test.

## Data Availability

The single-cell CITE-seq, VDJ-seq data and associated de-identified metadata have been uploaded into the NCBI Gene Expression Omnibus (GEO) data repository and are publicly available under the accession number GSE291290. The source code for data analysis is available on GitHub at https://github.com/yt723/Rhapsody-CITEseq. De-identified clinical data presented in this article will be made available upon reasonable request to the corresponding author.

## Abbreviations:

ACR: acute cellular rejection
ADT: antibody-derived tag
AMR: antibody-mediated rejection
CAV: cardiac allograft vasculopathy
CITE-seq: cellular indexing of transcriptomes and epitopes by sequencing
DSA: donor specific anti-HLA antibodies
GZMB: granzyme B
GZMK: granzyme K
HLA: human leukocyte antigen
HTx: heart transplant
IFNG: interferon gamma
ISHLT: International Society of Heart and Lung Transplant
LVEF: left ventricular ejection fraction
MFI: median fluorescence intensity
mRNA: messenger RNA
mTOR: mammalian target of rapamycin
NK: natural killer
PBMC: peripheral blood mononuclear cell
PRF: perforin
PRF1: perforin-1
RNA-seq: RNA sequencing
TCR: T cell receptor
Tem: effector memory T cell
Temra: effector memory re-expressing CD45RA T cell
TNF: tumor necrosis factor
UCSD: University of California, San Diego
UMAP: uniform manifold approximation and projection
UMI: unique molecular identifiers
VDJ-seq: variability, diversity, and joining segment sequencing
WNN: weighted nearest neighbor
