# Supplementary material for: Proinflammatory and cytotoxic CD38+HLA-DR+ effector memory CD8+ T cells are peripherally expanded in human cardiac allograft vasculopathy
Source: Am J Transplant. Author manuscript; Available in PMC 2026 Jan 7. (PMC12769763; doi:10.1016/j.ajt.2025.10.015)
Supplement: supp5 [file NIHMS2123269-supplement-supp5.pdf]

Supplemental Materials

Yuko Tada, MD, PhD[1]; Sujit Silas Armstrong Suthahar, BS[2]; Payel Roy, PhD[2,3]; Vasantika Suryawanshi, PhD[2]; Runpei Wu, BS[2]; Erpei Wang, PhD[2]; Felix Sebastian Nettersheim, MD[2,4]; Anusha Bellapu, MS[3]; Katarzyna Dobaczewska, BS[2]; Cheryl Kim, BS[2]; Florin Vaida, PhD[5]; Gerald P. Morris, MD, PhD[6]; Klaus Ley, MD[3]; Paul J. Kim, MD, MAS[1]
[1] Division of Cardiovascular Medicine, University of California, San Diego, San Diego, CA
[2] La Jolla Institute for Immunology, La Jolla, CA
[3] Immunology Center for Georgia, Augusta University, Augusta, GA
[4] Department of Cardiology, Faculty of Medicine and University Hospital Cologne, University of Cologne, Cologne, Germany
[5] Department of Family Medicine and Public Health, University of California, San Diego, San Diego, CA
[6] Department of Pathology, University of California, San Diego, San Diego, CA

*Corresponding Author
Paul J. Kim, MD, MAS
pjk017@health.ucsd.edu
Department of Medicine, UC San Diego Health
9452 Medical Center Drive, MC7411
La Jolla, CA92037, USA

Content

Supplementary Methods
Supplementary Table 1-7 (Table S1-7)
Supplementary Figure 1-14 (Figure S1-14)
Legends of Supplemental Excel File 1-4

Supplementary Methods

Sample Collection and Processing

Peripheral blood samples were collected in Cell Preparation Tubes (BD Biosciences) and mononuclear cells were isolated by density gradient centrifugation according to the manufacturer's instructions. Briefly, samples were centrifuged at 1500 g for 20 minutes at room temperature. The mononuclear cell layer was collected and washed with PBS two times. PBMCs were cryopreserved in the freezing media (5-10 million cells per tube; CryoStor CS10, STEMCELL technologies), frozen in -80°C overnight, and stored in liquid nitrogen until assay.

CITE-seq and VDJ-seq

Sample Preparation

The reagents used in the experiments are listed in **Table S1**. PBMCs were thawed in a 37°C water bath and contaminating red blood cells were lysed with red blood cell lysis buffer (eBioscience). After being washed, cells were suspended in Stain Buffer (BD Biosciences). Cell viability was measured on the Rhapsody scanner after being stained with Calcein AM (Invitrogen) and Draq7 (BD Biosciences). After cells from each patient were tagged with the Single-Cell Multiplexing Kit (BD Biosciences), cells from different samples (one CAV sample and two normal HTx samples for each batch/plate) were combined for hash-tagging. Samples were adjusted to contain up to 1 million cells and, after Fc blocking, incubated with AbSeq Ab-Oligos according to manufacturer's instructions. AbSeq Ab-Oligos master mix was created by mixing the reconstituted AbSeq

Immune discovery panel containing 30 antibodies (BD Biosciences) with 26 individual AbSeq antibodies (**Table S3**). After washing, single cells, adjusted to 7,500 cells per patient, were loaded on the BD Rhapsody Cartridge. Cells were lysed and mRNA, Ab-Oligos, and Sample Tags were hybridized with the barcoded oligos on the cell beads. Finally, 73.1% of the cells were captured by beads and retrieved. Cell viability at loading was 96.2±1.3% and cell multiplets rate on the Rhapsody scanner was 5.2±0.6% (**Table S4**).

Library Preparation and Sequencing

Libraries of mRNA, Sample Tag, AbSeq, and VDJ regions were prepared according to BD's library preparation protocol (23-22204-00). Cell capture beads underwent template switching using the template switch oligo (100μM 5'-TTT TTT TTT TTT TTT TTT TTT TTT TrGrGrG-3'), Klenow extension, and exonuclease I treatment. Two-step nested amplification was performed and followed by an index PCR (**Table S5**). PCR1 products were separated by double-sided AMPure bead purification into the shorter AbSeq and Sample Tag PCR products, and the longer targeted mRNA and VDJ PCR products. The AbSeq PCR1 product proceeded with index PCR. Targeted mRNA, VDJ, and Sample Tag PCR products underwent PCR2 amplification followed by index PCR. Quality of the final libraries were quantified using the Qubit dsDNA HS Kit and Agilent TapeStation high sensitivity D1000 screentape. mRNA, AbSeq, and SMK libraries were pooled together and sequenced by NovaSeq 6000 - S4 sequencer with 75 bp x 75 bp paired read. VDJ libraries were sequenced with the SP sequencer with 75 bp x 225 bp paired read. 400pM of the library was loaded, and 20-30% PhiX was included. Sequencing read depth was

calculated by 1,000 reads/Ab/cell for AbSeq, 10,000 reads per cell for mRNA, 1,000-1,200 reads per cell for sample tag, and 3,000 reads per cell for VDJ.

Data Analysis

Preprocessing of Data

FASTQ files obtained from the sequencing and FASTA references files were uploaded in the Seven Bridges Genomics pipeline (BD Biosciences) and processed as described in BD Single Cell Genomics Bioinformatics Handbook. Reads were first filtered to remove low quality pairs based on the read length, mean base quality score of the read and highest single nucleotide frequency observed across the bases of the read. R1 reads were annotated for cell labels and unique molecular identifiers (UMI). R2 reads were aligned to the reference panel sequences and annotated for mRNA, AbSeq and Sample tags. Reads with the same cell label, same UMI sequence, and same bioproduct were collapsed into a single raw molecule. After errors within the UMI were corrected and putative cells were identified, expression matrices of mRNA and antibody-derived tag (ADT) were obtained. Multiplets and undetermined cells were removed by the sample tag expression data. Low quality cells were removed by using the following thresholds: low numbers of genes per cell (<30), low numbers of UMIs per cell (<50), and the low complexity of RNA score (Log10 Genes per UMI < 0.60). Doublets were additionally removed using Doublet Finder (v2.0.3). Ultimately, 69,106 cells were retained for the downstream analysis.

Flow Cytometry

Cryopreserved PBMC were thawed and underwent red blood cell lysis if needed. After cell numbers were adjusted (2 million per sample), cells were stained with viability dye (1:2,000; Ghost Dye, TONBO). After Fc blocking (1:100; Human TruStain FcX Blocking Buffer, Biolegend), cells were stained with cell surface marker antibodies in 100μl of PBS containing 2% fetal bovine serum (FBS) at the optimized concentrations (**Table S6**). For cell surface marker staining, cells were first incubated with the anti-CCR7 antibody for 15 min at 37°C, and subsequently incubated with a cocktail of the other antibodies for 30 min at 4°C. Samples stained with fluorescence minus one (FMO) were prepared for determining the optimal thresholds for cell activation markers (CD38, HLA-DR, CD27, CCR7). Single color-stained cells were used for unmixing. For intracellular staining (ICS) of cytokines and cytotoxic enzymes, after cell surface staining, cells underwent fixation and permeabilization (eBioscience), and were incubated with antibodies in the permeabilization buffer at optimized concentrations for 45 minutes at room temperature. For ICS of inflammatory cytokines (IFNG, TNF), cells were stimulated before staining. For stimulation, cells were plated at a concentration of $1x10^6$ cells per well in 96 well plates. After incubation overnight at 37℃, PMA/ionomycin stimulation cocktail (eBioscience) was added to each well. Two hours later, the protein transport inhibitor cocktail (eBioscience) was added, and cells were incubated for an additional 4 hours. Only the protein transport inhibitor cocktail was added to unstimulated control groups and cells were incubated for the same period. Data was acquired using the Cytek Aurora spectral flow cytometer (Cytek® Biosciences). Flow cytometry data was analyzed using FlowJo (v10.9.0; BD Biosciences and FlowJo LLC).

Histological Immunofluorescence Staining

Formalin-fixed paraffin-embedded tissues were deparaffinized and antigen retrieval was performed by heating samples in the citrate buffer (pH 6.0) in the Antigen Retrieval Chamber (BioCare Medical). To reduce tissue autofluorescence, slides were placed in a transparent reservoir containing 4.5% H_2O_2 and 24 mM NaOH in PBS and illuminated with white light for 60 minutes followed by 365 nm light for 30 minutes at room temperature. Two immunofluorescence panels targeting CD8/CD38/HLA-DR or CD4/CD38/HLA-DR proteins were used for staining. After blocking with 5% normal donkey serum (0.3% triton X in PBS) and a subsequent Avidin/Biotin blocking (SP-2001; Vector Laboratories), samples were incubated with primary antibodies; mouse anti-CD8 (clones C8/144B and MA5-13473), goat anti-CD4 (polyclonal, AF-379) and Biotin-conjugated rabbit anti-CD38 (clones EPR4106 and ab201498) with optimized concentration at 4°C overnight. After washing, slides were incubated with secondary antibodies conjugated with fluorescence (AF568-conjugated goat anti-mouse IgG1, #A21124, Invitrogen; AF555-conjugated donkey anti-goat IgG, #A32816, Invitrogen; AF488-conjugated streptavidin, #S11223, Invitrogen) for 1 hour at room temperature. Samples were then stained with rabbit AF647 anti-HLA-DR antibodies (clones EPR3692 and ab307776) at 4°C overnight. Slides were counterstained with Hoechst 33342 (Invitrogen). Stained slides were scanned by Zeiss Axioscan Z1 slide scanner and analyzed using QuPath (v.0.5.1). For quantification of cell numbers in the intima, images were snapshot at the same magnification (x20). Five images per patient obtained at the equivalent locations were used to compare the CD4 and CD8 panels. Images from different channels were stacked and ROIs were placed in the intima using ImageJ. Cells

in the intima were counted using the ImageJ multi-point tool. Total cell number was determined based on the number of nuclei in the region of interest.

Supplementary Tables S1-S7

Table S1. Major resources table

Reagent	Vendor	Catalog #
BD Vacutainer CPT	BD	362760
PBS	Fisher Scientific	10010049
Stain buffer	BD	564904
RBC Lysis Buffer (1x)	eBioscience	00-4333-57
DRAQ7	BD	564904
Calcein AM	Thermo Fisher	C1430
DMSO	Thermo Fisher	D12345
Fc Block	BD	564220
SMK	BD	633781
AbSeq Immune discovery panel	BD	625970
Rhapsody Reagent Kit	BD	633731
Human Immune Response Panel	BD	633750
Targeted mRNA and AbSeq Amplification Kit	BD	633774
RhapsodyTM cDNA Kit	BD	633773
AMPure XP beads	Beckman Coulter	A63880
D1000 ScreenTape	Agilent	5067-5584
D1000 Sample Buffer	Agilent	5067-5603
Qubit Reagent Kit	Thermo Fisher	Q33231
NovaSeq SP 100 Cycle Kit	Illumina	20028041
NovaSeq S4 200 Cycle Kit	Illumina	20028313
Agencourt AMPure XP magnetic beads	Beckman Coulter	A63880
Tris-Tween20	Teknova	T1485
Template switch oligo	Integrated DNA Technologies	-
Klenow Fragment	New England Biolabs	M0212L
DNA Suspension Buffer	Teknova	T0221
Intracellular fixation/permeabilization buffer	eBioscience	88-8240-00
PMA/ionomycin stimulation cocktail	eBioscience	00-4970-93
Protein transport inhibitor cocktail	eBioscience	00-4980-03

Table S2. Software packages and versions used in the analysis

Software package	Version
Seurat	5.2.0
SingleR	2.6.0
celldex	1.14.0
pheatmap	1.0.12
ComplexHeatmap	2.20.0
ggplot2	3.5.1
sccomp	1.8.0
edgeR	4.2.2
clusterProfiler	4.12.6
GOAT	1.1.2
destiny	3.18.0
iNEXT	3.0.1
vegan	2.6-8
FlowJo	10.9.0
QuPath	0.5.1
GraphPad Prism	10

Table S3. AbSeq antibodies

Specificity	Clone	Catalog #
CD11b	M1/70	940008
CD11c	B-LY6	IDP
CD123 (IL-3RA)	7G3	940020
CD126 (IL-6R)	M5	940090
CD127 (IL-7R)	HIL-7R-M21	IDP
CD137	4B4-1	IDP
CD14	MPHIP9	IDP
CD141	1A4	940079
CD142	HTF-1	940280
CD152 (CTLA-4)	BNI3	940034
CD154	TRAP1	940053
CD16	3G8	IDP
CD163	GHI/61	940058
CD183 (CXCR3)	1C6/CXCR3	IDP
CD184 (CXCR4)	12G5	940056
CD185 (CXCR5)	RF8B2	IDP
CD19	SJ25C1	IDP
CD192 (CCR2)	1D9	940286
CD194 (CCR4)	1G1	940047
CD195 (CCR5)	2D7/CCR5	940050
CD196 (CCR6)	11A9	IDP
CD197 (CCR7)	LS132.1D9	IDP
CD2	RPA-2.10	940046
CD20	2H7	940016
CD206	19.2	940068
CD223 (LAG-3)	T47-530	940080
CD25	2A3	IDP
CD27	M-T271	IDP
CD3	UCHT1	IDP
CD36	CLB-IVC7	940224
CD38	HIT2	940013
CD4	SK3	IDP
CD45RA	HI100	IDP
CD45RO	UCHL1	940022

CD56	NCAM16.2	IDP
CD69	FN50	940019
CD8	SK1	IDP
CD86	2331(FUN-1)	940025
CD9	M-L13	940078
HLA-DR (CD74)	G46-6	IDP
CD24	ML5	940028
CD33	WM53	940031
IgM	G20-127	IDP
IgD	IA6-2	IDP
CD273	MIH18	940071
CD274	MIH1	940035
CD279	MIH4	IDP
CD28	L293	IDP
CD62L	DREG-56	IDP
CD134	ACT35	IDP
CD161	HP-3G10	IDP
CD186 (CXCR6)	13B 1E5	IDP
CD272	J168-540	IDP
CD278	DX29	IDP
CD357 (GITR)	V27-580	IDP
CD366 (Tim3)	7D3	IDP

IDP=BD AbSeq Immune Discovery Panel (Catalog #: 625970)

Table S4. Batch information and sample viability

		Initial viability	Viability at loading	multiplet rate
Batch 1	CAV1	94.5%	96.5%	6.4%
	Normal_HTx1_1	98.0%		
	Normal_HTx1_2	97.5%		
Batch 2	CAV2	96.7%	96.9%	5.2%
	Normal_HTx2_1	94.4%		
	Normal_HTx2_2	96.1%		
Batch 3	CAV3	97.9%	95.8%	5.0%
	Normal_HTx3_1	95.6%		
	Normal_HTx3_2	98.8%		
Batch 4	CAV4	73.0%	93.7%	4.6%
	Normal_HTx4_1	97.3%		
	Normal_HTx4_2	97.4%		
Batch 5	CAV5	95.3%	97.4%	4.7%
	Normal_HTx5_1	97.3%		
	Normal_HTx5_2	97.4%		
Batch 6	CAV6	98.6%	96.5%	5.5%
	Normal_HTx6_1	98.0%		
	Normal_HTx6_2	96.2%		
Average		95.6±5.7%	96.2±1.3%	5.2±0.6%

Mean (±SD) values are shown. Cell number at loading was adjusted to 7,500 per patient.

Table S5. Polymerase chain reaction (PCR) primers for human T cell and B cell

**Human T cell PCR1 primers (5'-3' sequence)**	
TRAC_N1	*CTGGAATAATGCTGTTGTTGAAGG*
TRBC_N1	*AGCCCGTAGAACTGGACTT*
TRDC_N1	*CTTCAAAGTCAGTGGAGTGCA*
TRGC_N1	*CACCGTTAACCAGCTAAATTTCATG*
**Human T cell PCR2 primers (5'-3' sequence)**	
TRAC_N2	*CAGACGTGTGCTCTTCCGATCTATCAAAATCGGTGAATAGGCAGAC*
TRBC_N2	*CAGACGTGTGCTCTTCCGATCTGATCTCTGCTTCTGATGGCTCA*
TRDC_N2	*CAGACGTGTGCTCTTCCGATCTATATCCTTGGGGTAGAATTCCTTC*
TRGC_N2	*CAGACGTGTGCTCTTCCGATCTGGGAAACATCTGCATCAAGTTG*
**Human B cell PCR1 primers (5'-3' sequence)**	
IGHA_N1	*CACAGTCACATCCTGGCT*
IGHD_N1	*GATCTCCTTCTTACTCTTGCTGG*
IGHE_N1	*CGCTGAAGGTTTTGTTGTCG*
IGHG_N1	*TGTTGCTGGGCTTGTGAT*
IGHM_N1	*CGTTCTTTTCTTTGTTGCCGT*
IGKC_N1	*TTTGTGTTTCTCGTAGTCTGCT*
IGLC_N1	*TGTAGCTTCTGTGGGACTTC*
**Human B cell PCR2 primers (5'-3' sequence)**	
IGHA_N2	*CAGACGTGTGCTCTTCCGATCTCTTTCGCTCCAGGTCACACT*
IGHD_N2	*CAGACGTGTGCTCTTCCGATCTTGTCTGCACCCTGATATGATGG*
IGHE_N2	*CAGACGTGTGCTCTTCCGATCTGTCAAGGGGAAGACGGATG*
IGHG_N2	*CAGACGTGTGTGCTCTTCCGATCTAAGTAGTCCTTGACCAGGCA*
IGHM_N2	*CAGACGTGTGCTCTTCCGATCTACAGGAGACGAGGGGAAAA*
IGKC_N2	*CAGACGTGTGCTCTTCCGATCTTCAGATGGCGGGAAGATGAA*
IGLC_N2	*CAGACGTGTGCTCTTCCGATCTACCAGTGTGGCCTTGTTG*

Table S6. Flow cytometry antibodies

Target	Clone	Fluorescence	Catalog #	Dilution
Ghost dye	-	BV510	SKU-13-0870	1:2000
HLA-DR	L243	BUV395	564040	1:33
CD38	HB7	BUV737	612824	1:50
CD19	HIB19	BV421	302233	1:50
CD16	3GB	BV570	302036	1:50
CD197	G043H7	BV711	353227	1:25
CD4	OKT4	BV785	317442	1:50
CD45RA	HI100	APC	304112	1:50
CD3	UCHT1	Alexa Fluor 700	304112	1:50
CD8	RPA-T8	APC-Cy7	301016	1:50
CD14	M5E2	FITC	301804	1:50
CD161	HP-3G10	PE	339904	1:50
CD27	H-T271	PE/Dazzle594	356422	1:50
CD56	5.1H11	PE-Cy5	362516	1:50
CD45RO	UCHL1	PE-Cy7	304230	1:100
IFNG	4S.B3	BV650	502538	1:25
TNF	Mab11	FITC	502906	1:25
GZMK	GM26E7	FITC	370508	1:33
GZMB	GB11	RB780	568705	1:33
PRF	dG9	Pacific Blue	308118	1:33

Table S7. Flow cytometry cell proportion analytical results

		HC (n=11)	Normal HTx (n=12)	High grade CAV (n=11)	p value*
**Major cell type (%CD45+ Cell)**					
Classical monocyte		5.7 (3.2-7.4)	9.9 (4.5-20.1)	9.3 (5.2-26.4)†	0.064
Intermediate monocyte		0.5 (0.4-0.5)	0.7 (0.4-1.7)	0.9 (0.6-2.2)†	**0.047**
Nonclassical monocyte		0.8 (0.5-1.6)	1.6 (0.9-3.7)	1.4 (0.9-1.6)	0.153
Dendritic cell		0.9 (0.8-1.3)	2.5 (1.1-4.0)	2.7 (1.0-4.6)	0.115
B		8.1 (6.2-11.7)	5.1 (3.6-9.9)	6.1 (2.8-11.3)	0.488
T		63.9 (57.9-68.8)	49.1 (38.2-71.7)	52.5 (31.4-68.5)	0.260
NK		14.2 (10.5-23.7)	12.7 (11.6-21.4)	16.7 (11.2-24.3)	0.862
**T cell subpopulation**					
CD8 T (%T cell)		33.5 (22.4-33.9)	47.3 (38.8-58.5)†	41.3 (30.8-46.5)†	**0.004**
CD4 T (%T cell)		60.6 (56.0-70.8)	45.9 (36.3-55.9)†	51.8 (45.4-59.4)†	**0.015**
CD4/CD8T ratio		1.8 (1.7-3.2)	1.0 (0.6-1.4)†	1.3 (1.0-1.9)†	**0.012**
**CD8 T cell subpopulation (%CD8 T cell)**					
CCR7+CD27+ (C3)		55.7 (25.5-65.4)	17.0 (10.4-26.5)†	13.2 (9.2-17.8)†	**<0.001**
CCR7-CD27+		25.4 (11.5-33.3)	18.1 (13.5-22.4)	23.2 (17.3-28.9)	0.196
	CD38-HLA-DR- (C7)	14.9 (8.8-22.8)	10.5 (8.4-13.2)	7.7 (3.3-16.4)†	0.080
	CD38-HLA-DR+ (C2)	2.9 (1.6-4.0)	3.1 (2.2-6.8)	4.6 (1.5-10.3)	0.426
	CD38+ HLA-DR+ (C4)	2.2 (1.6-2.7)	1.5 (0.9-3.6)	3.3 (2.4-9.8)†‡	**0.025**
CCR7-CD27-		6.9 (4.3-23.9)	62.4 (46.8-66.9)†	58.8 (48.4-62.5)†	**<0.001**
	CD56+ (C5)	1.9 (0.6-4.1)	15.0 (4.0-21.3)†	13.2 (5.8-22.0)†	**0.002**
	CD56- (C1)	5.1 (3.3-13.8)	40.4 (28.5-52.4)†	35.5 (25.9-52.8)†	**<0.001**
	CD38+ HLA-DR+	7.1 (5.1-11.2)	9.7 (6.0-23.9)	21.6 (8.5-30.5)†	0.057
MAIT (C6)		8.2 (3.5-11.4)	1.3 (0.6-2.5)†	2.1 (0.4-2.5)†	**0.001**
**CD4 T cell subpopulation (%CD4 T cell)**					
CCR7+CD27+		81.8 (75.6-87.4)	58.3 (40.8-74.5)†	35.9 (24.8-70.3)†	**0.001**
CCR7-CD27+		13.8 (10.4-18.6)	13.2 (7.2-18.3)	13.1 (10.5-18.3)	0.971

CCR7-CD27-	3.2 (2.6-5.5)	24.2 (10.6-36.6)†	38.7 (16.1-50.9)†	**<0.001**

Median (IQR) values are shown in the table.
p value*: Kruskal-Wallis test
Post-hoc pairwise comparison (Dunn’s test): (†) unadjusted p<0.05 compared to HC, (‡) unadjusted p<0.05 compared to normal HTx

SUPPLEMENTARY FIGURES

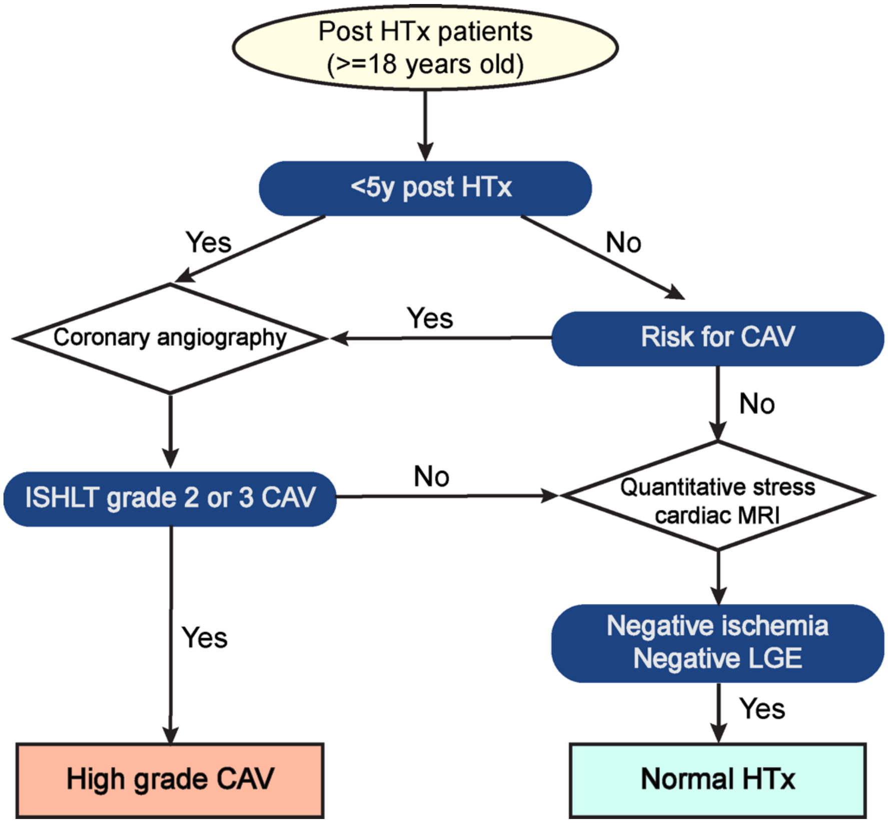

Figure S1. Patient recruitment strategy.

The flow chart shows the inclusion criteria for normal HTx and high-grade CAV in this study. Patients < 5 years post HTx or those with higher risk for CAV (i.e., prior diagnosis of ISHLT grade 1 CAV or greater, history of antibody mediated rejection, history of positive DSA testing in the past year, or signs/symptoms of cardiac allograft dysfunction) underwent coronary angiography for screening of CAV. Patients ≥ 5 years post HTx and at low risk for CAV were evaluated non-invasively. Low risk patients were defined as ISHLT grade 0 CAV on the prior annual coronary angiogram, negative DSA testing in the past year, left ventricular ejection fraction higher than 50% and without signs/symptoms of cardiac allograft dysfunction. A subset of these patients were referred for quantitative stress cardiac MRI as part of a separate multi-center study.[18] A subset of HTx patients <5y post HTx and without ISHLT grade 2 or 3 CAV were also referred for quantitative stress cardiac MRI as part of the multi-center study. HTx patients with normal stress cardiac MRI results and without prior diagnosis of ISHLT grade 1-3 CAV were included in the normal HTx group. Patients who were diagnosed with ISHLT grade 2 or 3 CAV by coronary angiography were approached for enrollment in the high-grade CAV group.

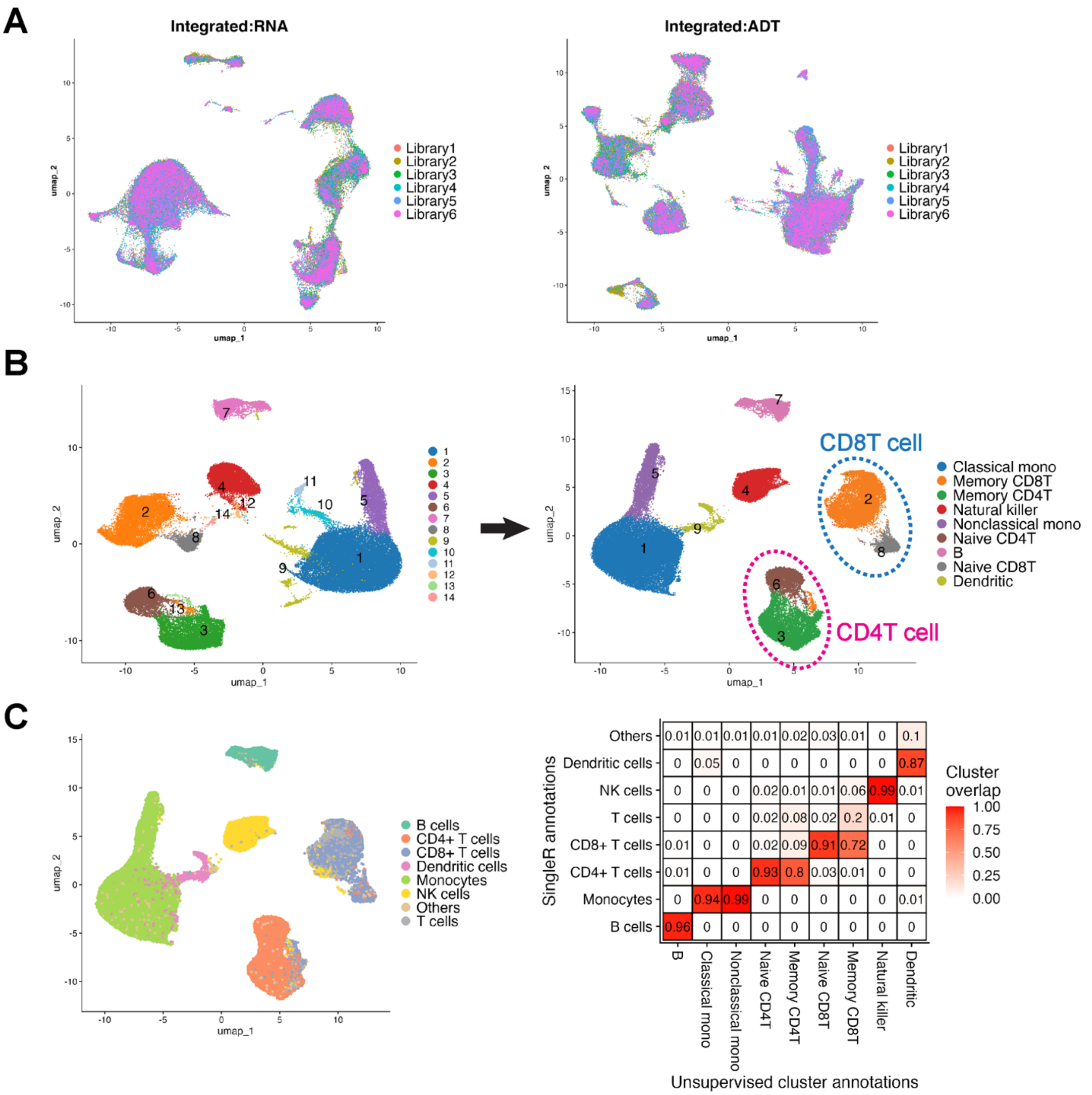

Figure S2. Major cell type clustering.

A. Batch effect was taken into account using canonical correlation analysis. Corrected RNA (left) and ADT (right) data, integrated separately, are shown in the uniform manifold approximation and projection (UMAP). **B.** Major cell type clustering of peripheral blood mononuclear cells (PBMCs) was performed using the CITE-seq expression data of the 18 antibody-derived tag (ADT) markers (CCR7, CD2, CD3, CD4, CD8, CD9, CD11b, CD11c, CD14, CD16, CD19, CD20, CD36, CD38, CD45RA, CD45RO, CD56, HLA-DR) across all samples (n=18). From the initial 14 clusters (left UMAP), 3 doublet clusters (clusters 9, 12, and 14) were removed. Remaining cells were re-clustered and the final 9 major cell types were obtained (right UMAP). CD8^{+} T cell clusters and CD4^{+} T cell clusters

used for targeted clustering are annotated on the map. **C.** Reference-based cell annotations using the transcriptomic data and the Monaco immune database closely approximate the initial major immune cell cluster assignments (left UMAP). The right heatmap demonstrates high consistency of major immune cell cluster annotations between the unsupervised and reference-based methods. The number in each tile represents the proportion of cells in the unsupervised cluster assigned to the specific SingleR cluster (column sum = 1.0).

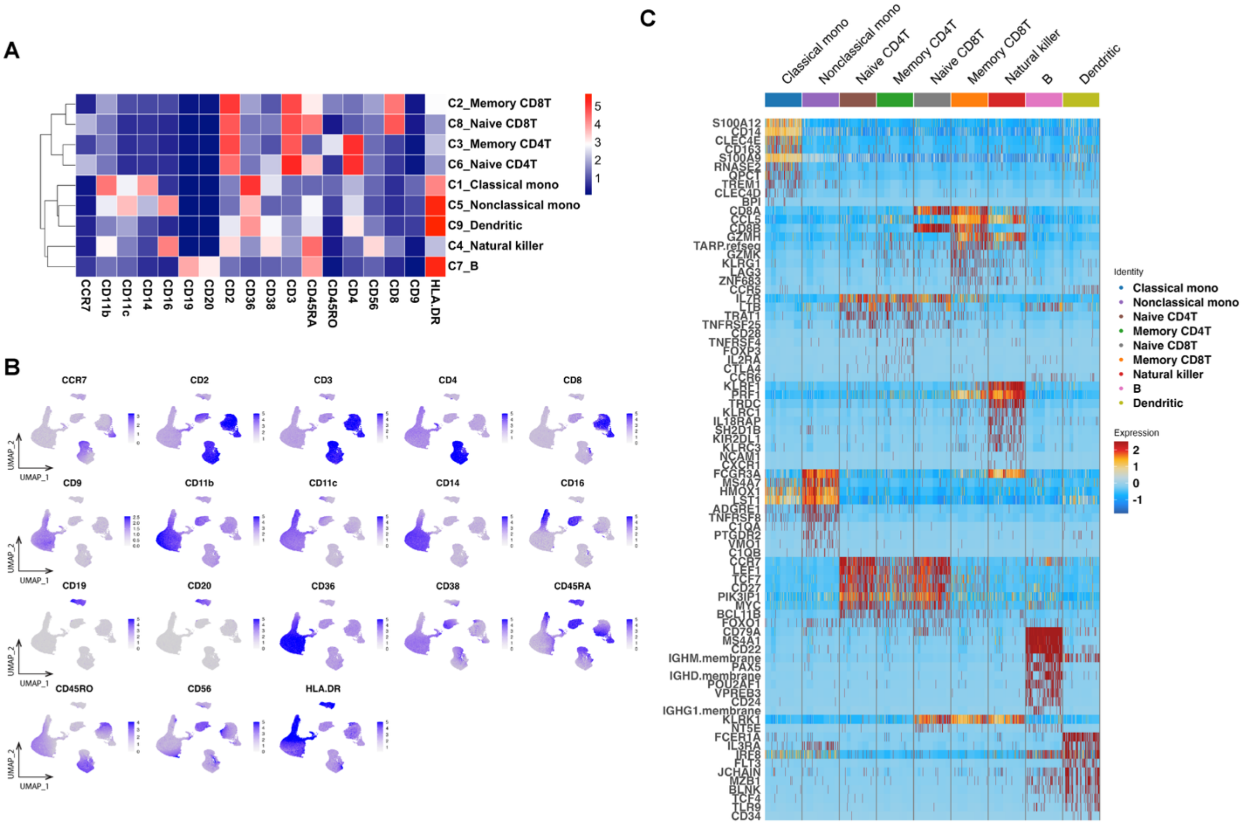

Figure S3. Surface markers and gene expression of the major immune cell types.

A. Heatmap showing the average centered log ratio normalized expression of the 18 ADT markers in each of the 9 major immune cell type clusters obtained from CITE-seq data. **B.** Feature plots of the 18 ADT markers for the 9 major immune cell type clusters. **C.** Heatmap comparing cell-level expression of top differentially expressed genes among the major immune cell type clusters. Heatmap was created using the ComplexHeatmap in R and cells were down-sampled to equal numbers across the major cell types.

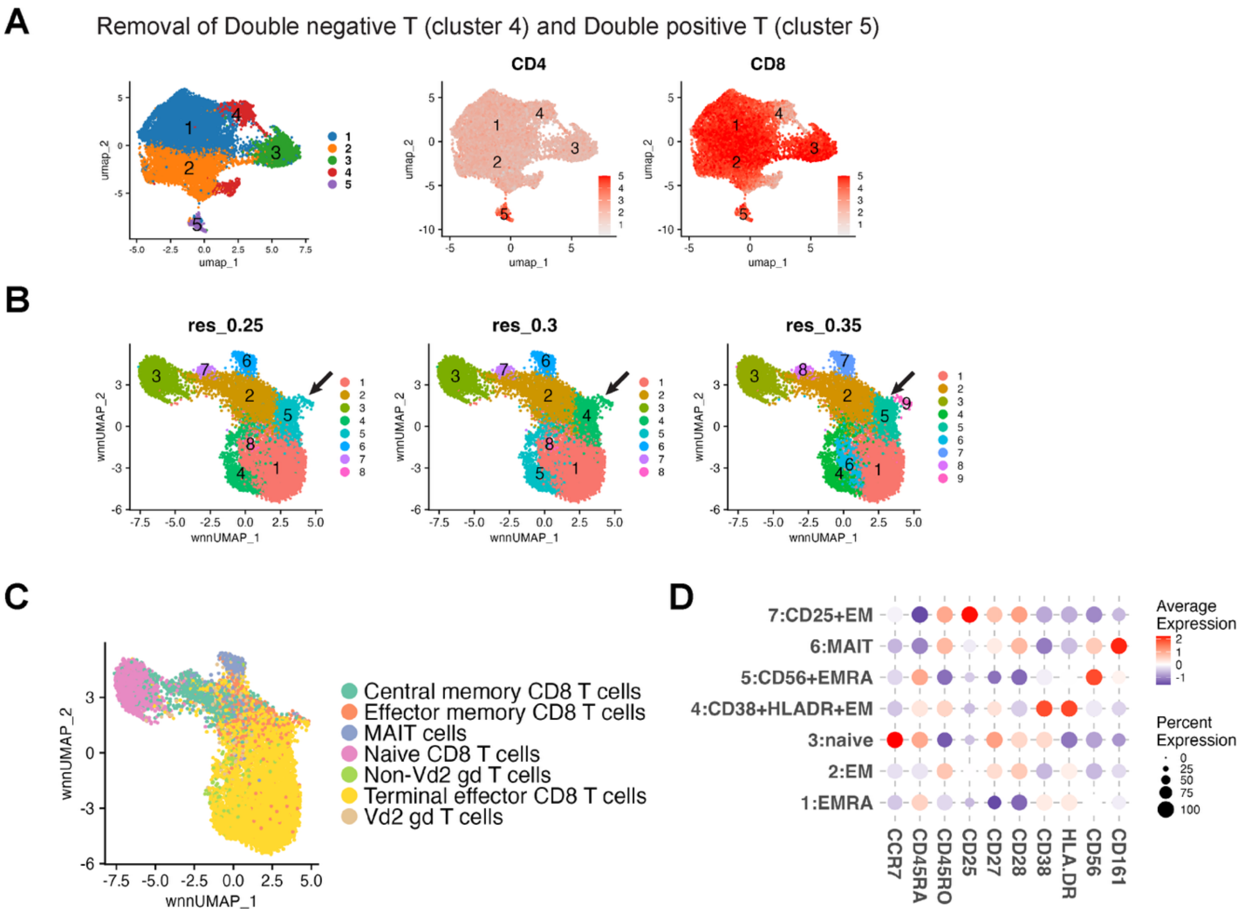

Figure S4. Characterization of CD8$^+$ T cell subclusters.

A. The double negative (cluster 4) and double positive (cluster 5) T cell clusters were identified after clustering naïve and memory CD8$^+$ T cells (left UMAP). Featured UMAPs (right) show CD4 and CD8 expressions. Those 2 clusters were removed to obtain single positive CD8$^+$ T cells. **B.** Different resolutions (0.25, 0.3, 0.35) were tested for WNN unsupervised clustering of CD8$^+$ T cells. The CD38$^+$HLA-DR$^+$ Tem cluster (black arrows) was consistently identified despite changes in the resolutions. Resolution 0.3 was selected relevant to known functional CD8 T cell populations, including naive, memory, and TEMRA clusters. Cluster 8 was removed due to its small size (n=48). **C.** Reference based annotation using the Monaco immune database is shown in the UMAP for comparison. **D.** Expression of the representative surface proteins for calling CD8$^+$ T cell clusters are shown in the dot plot. Color scale represents the scaled normalized expression of ADT.

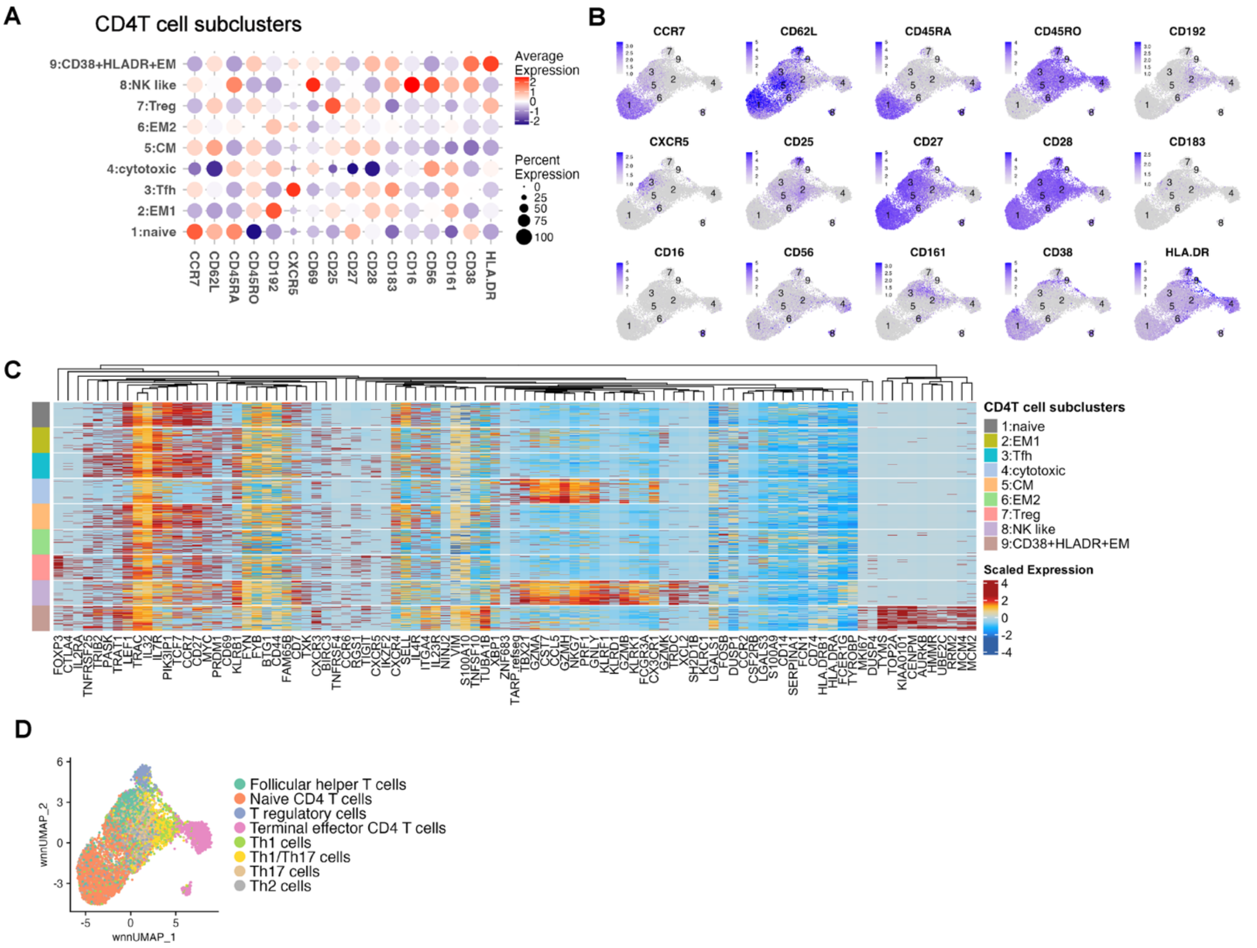

Figure S5. Characterization of CD4⁺ T cell subclusters.

CD4⁺ T cell clusters were obtained by WNN analysis of the CITE-seq data. **A** and **B.** Expression of the representative surface proteins for calling cell types are shown in the dot plot (**A**) and feature plots (**B**). **C.** Heatmap comparing cell-level expression of differentially expressed genes among the CD4⁺ T cell subclusters. Cells were down-sampled to equal numbers across the subclusters. For **A** and **C**, color scale represents the scaled normalized expression values of ADT and gene expression. **D.** Reference based annotation using the Monaco immune database is shown in the UMAP for comparison.

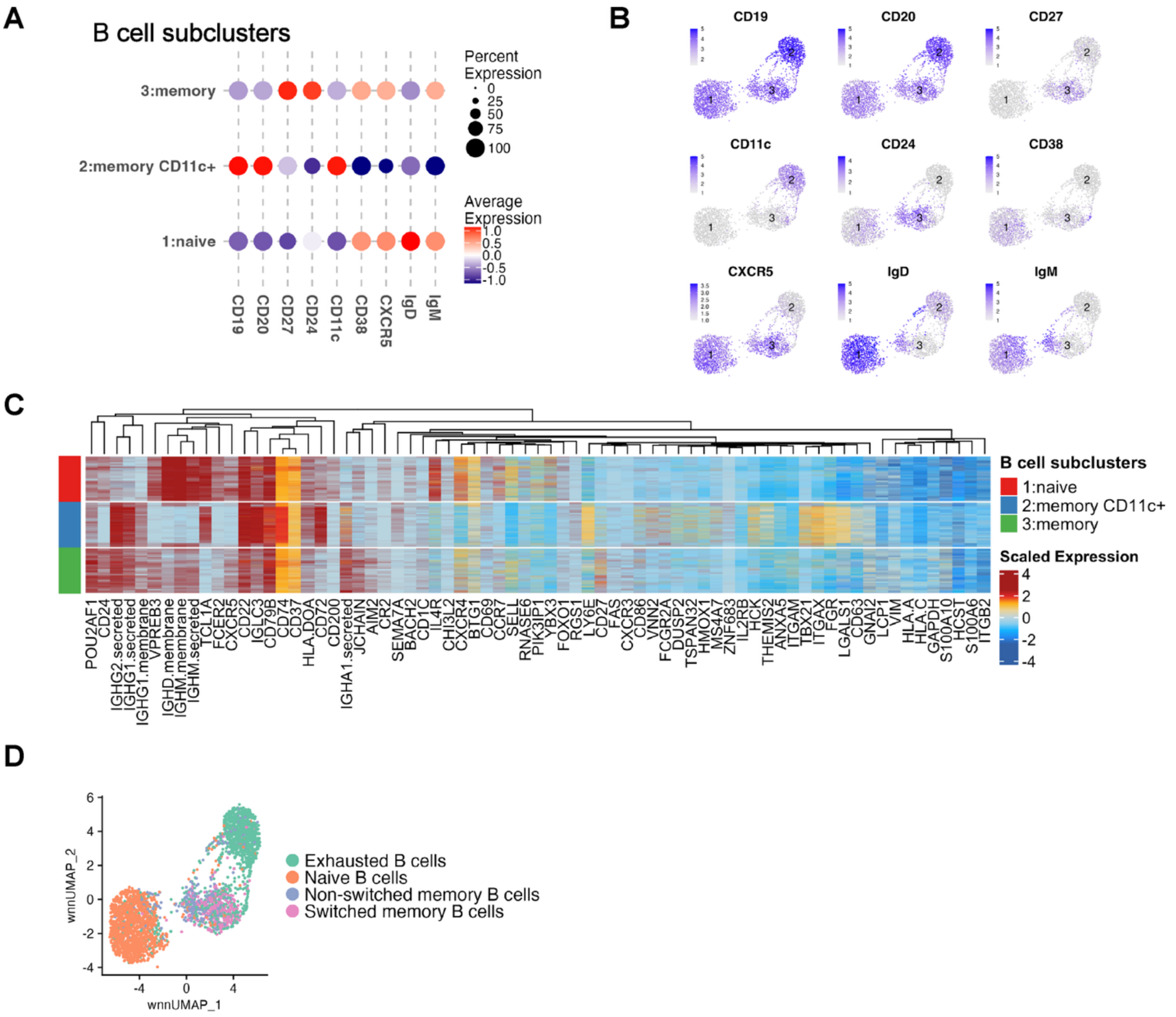

Figure S6. Characterization of B cell subclusters.

B cell clusters were obtained by WNN analysis of the CITE-seq data. **A** and **B.** Expression of the representative surface proteins for calling cell types are shown in the dot plot (**A**) and feature plots (**B**). **C.** Heatmap comparing cell-level expression of differentially expressed genes among the B cell subclusters. Cells were down-sampled to equal numbers across the subclusters. For **A** and **C**, color scale represents the scaled normalized expression values of ADT and gene expression. **D.** Reference based annotation using the Monaco immune database is shown in the UMAP for comparison.

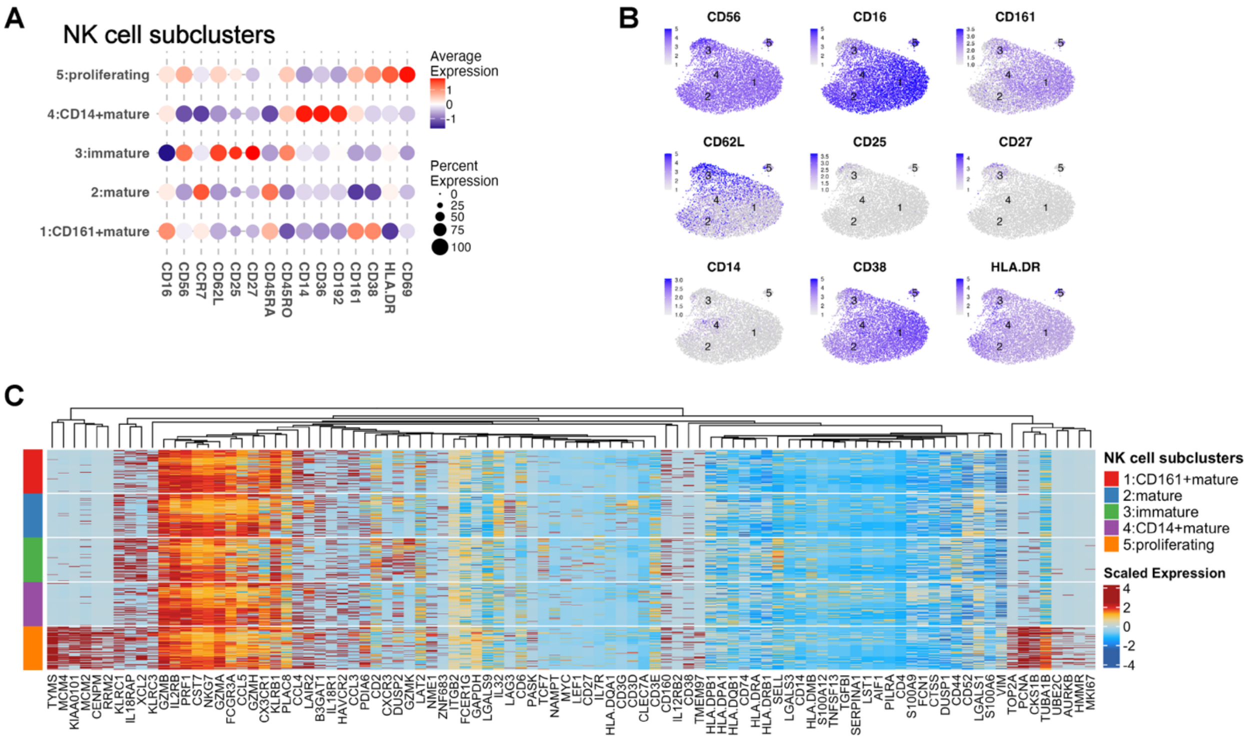

Figure S7. Characterization of NK cell subclusters.

NK cell clusters were obtained by WNN analysis of the CITE-seq data. **A** and **B.** Expression of the representative surface proteins for calling cell types are shown in the dot plot (**A**) and feature plots (**B**). **C.** Heatmap comparing cell-level expression of differentially expressed genes among the NK cell subclusters. Cells were down sampled to equal numbers across the subclusters. For **A** and **C**, color scale represents the scaled normalized expression values of ADT and gene expression.

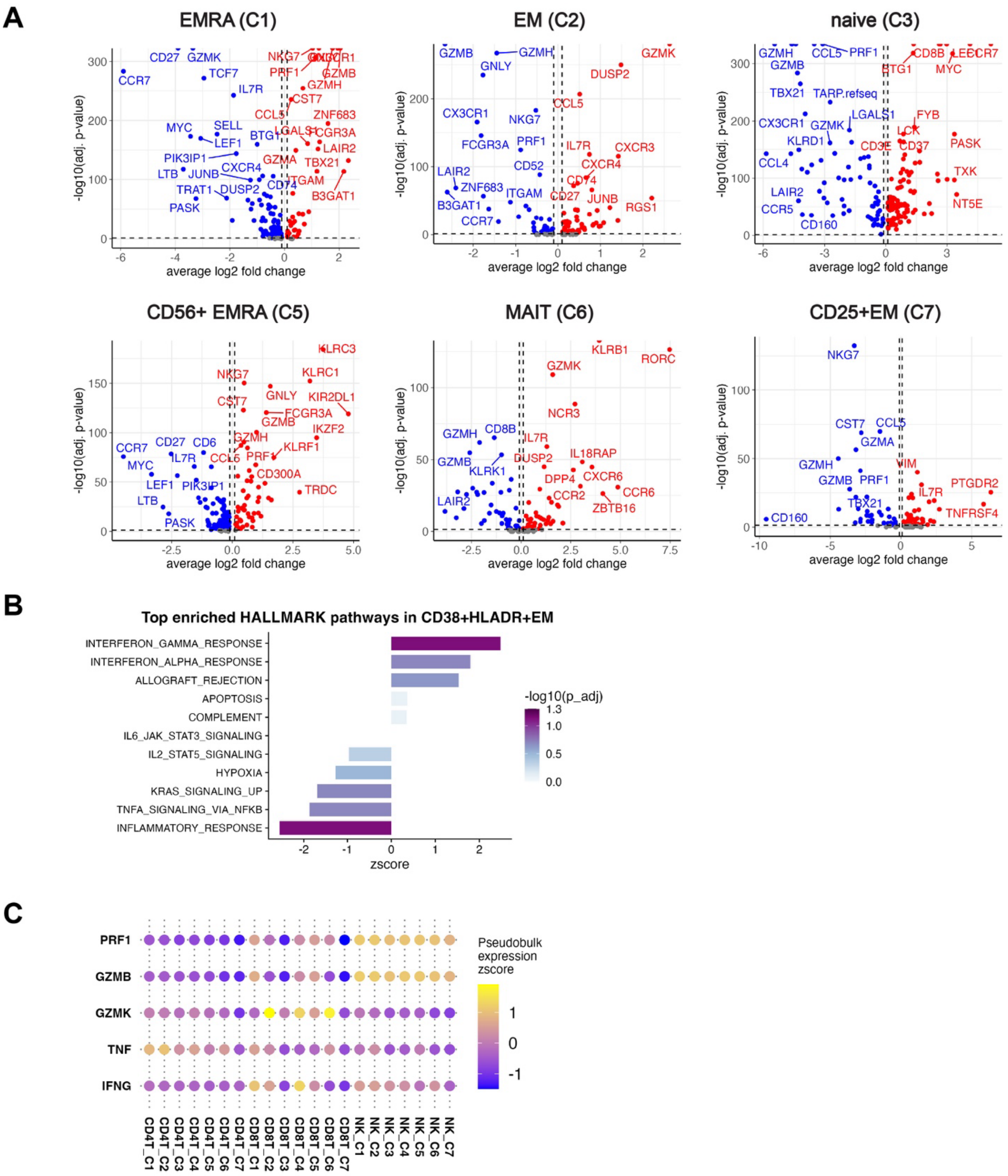

Figure S8. Differential gene expression by CD8$^+$ T cell subclusters

A. Volcano plots show the differentially expressed genes of each CD8$^+$ T cell cluster other than that of the CD38$^+$HLA-DR$^+$ CD8$^+$ Tem, which is shown in Figure 3C. P-values were adjusted by including the individual PBMC sample as a covariate and using the Benjamini-Hochberg procedure for FDR-adjusted p-values. **B.** The barplot shows the top

most enriched Hallmark pathways in the CD38$^+$HLA-DR$^+$ CD8$^+$ Tem cluster determined by the gene set ordinal association test. x-axis represents the z-score and the color scale in the legend represents the log-transformed adjusted p-value. **C.** Dot plots comparing IFNG expression among all T and NK cell clusters showed the highest IFNG expression in the CD38$^+$HLA-DR$^+$ CD8$^+$ Tem cluster (CD8T_C4).

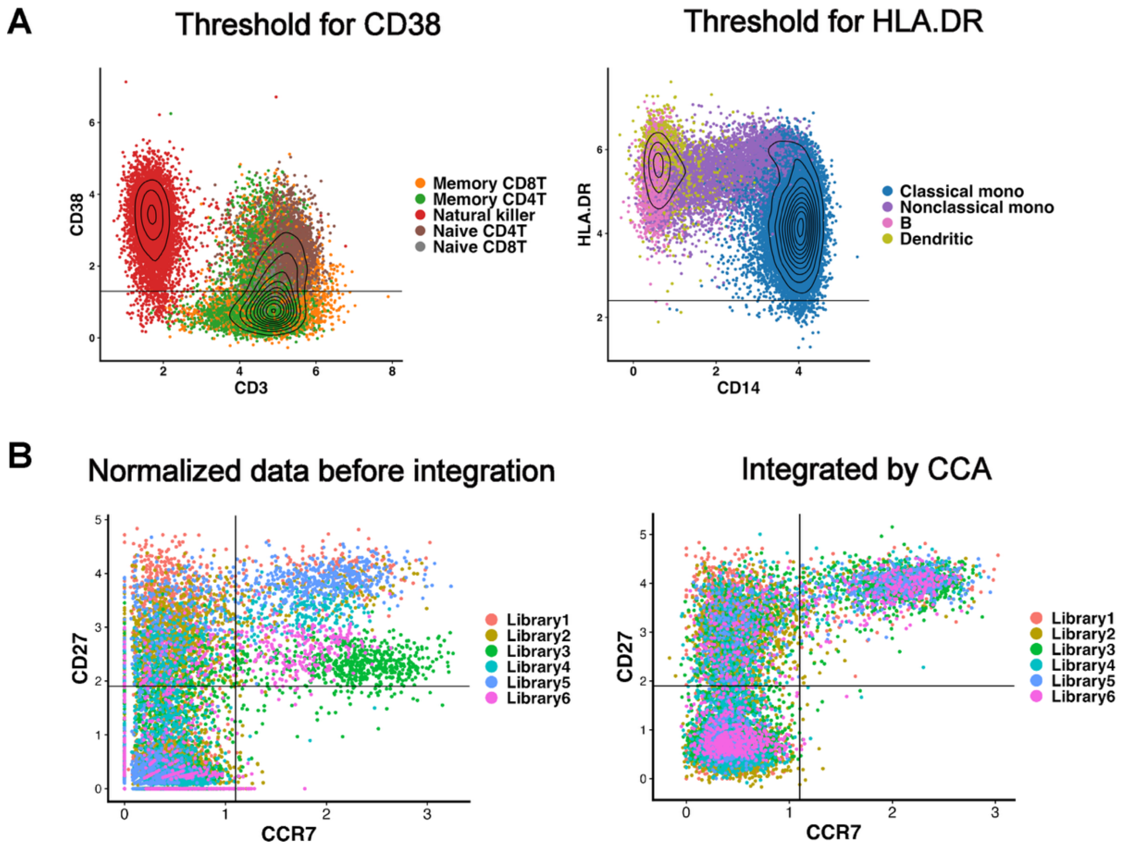

Figure S9. Thresholds for defining positive CD38 and HLA-DR expression.

A. Feature scatter plots show the expression of CD38 (left) and HLA-DR (right) for major cell types. Thresholds to define positive CD38 and HLA-DR expression were determined based on the expressions of these markers by the major cell types known to positively express CD38 (natural killer cells) and HLA-DR (monocytes, B cells, dendritic cells). **B.** The centered log-ratio normalized data (left) was first integrated using canonical correlation analysis (right) prior to analysis based on thresholding ADT expression.

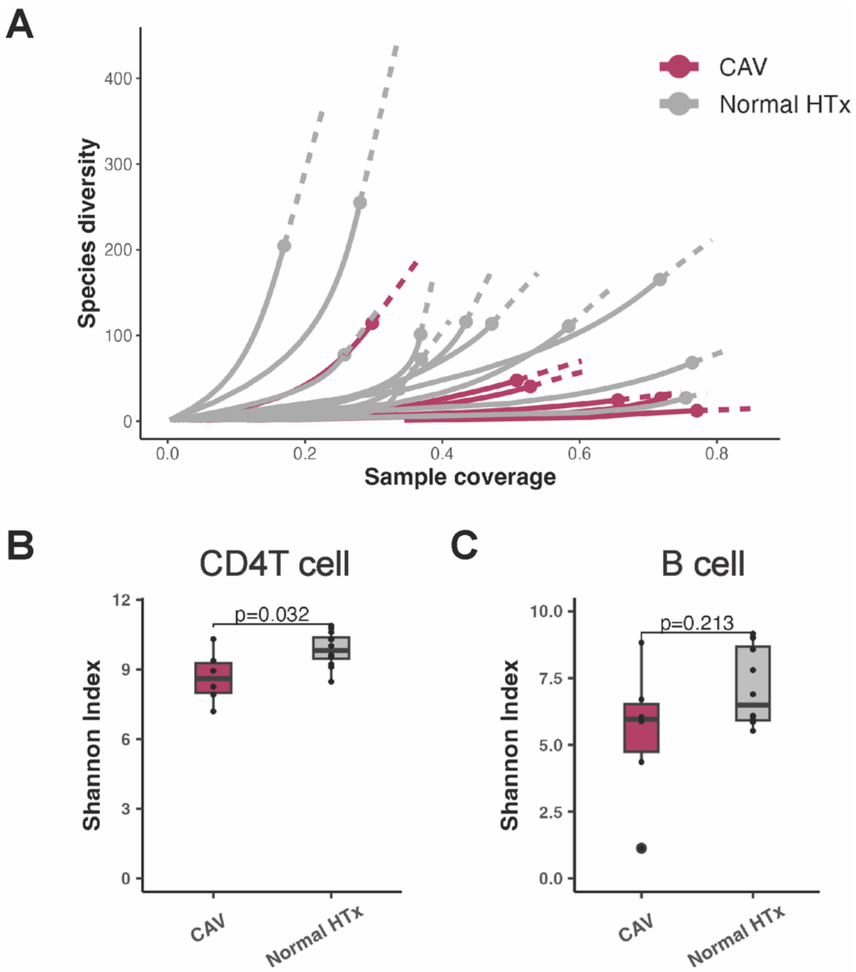

Figure S10. T cell and B cell receptor repertoire analysis.

A. Paired CDR3 alpha and beta chain T cell receptor (TCR) repertoire diversity of $CD8^{+}$ T cells was estimated by bootstrapped rarefaction/extrapolation curve of Hill's number. Sample diversity against sample coverage curve of each sample shows decreased species diversity in the high-grade CAV compared to normal HTx patients. **B** and **C.** The T cell or B cell receptor Shannon diversity index was compared between high-grade CAV and normal HTx (Wilcoxon rank-sum test) for $CD4^{+}$ T cells (**B**) and B cells (**C**).

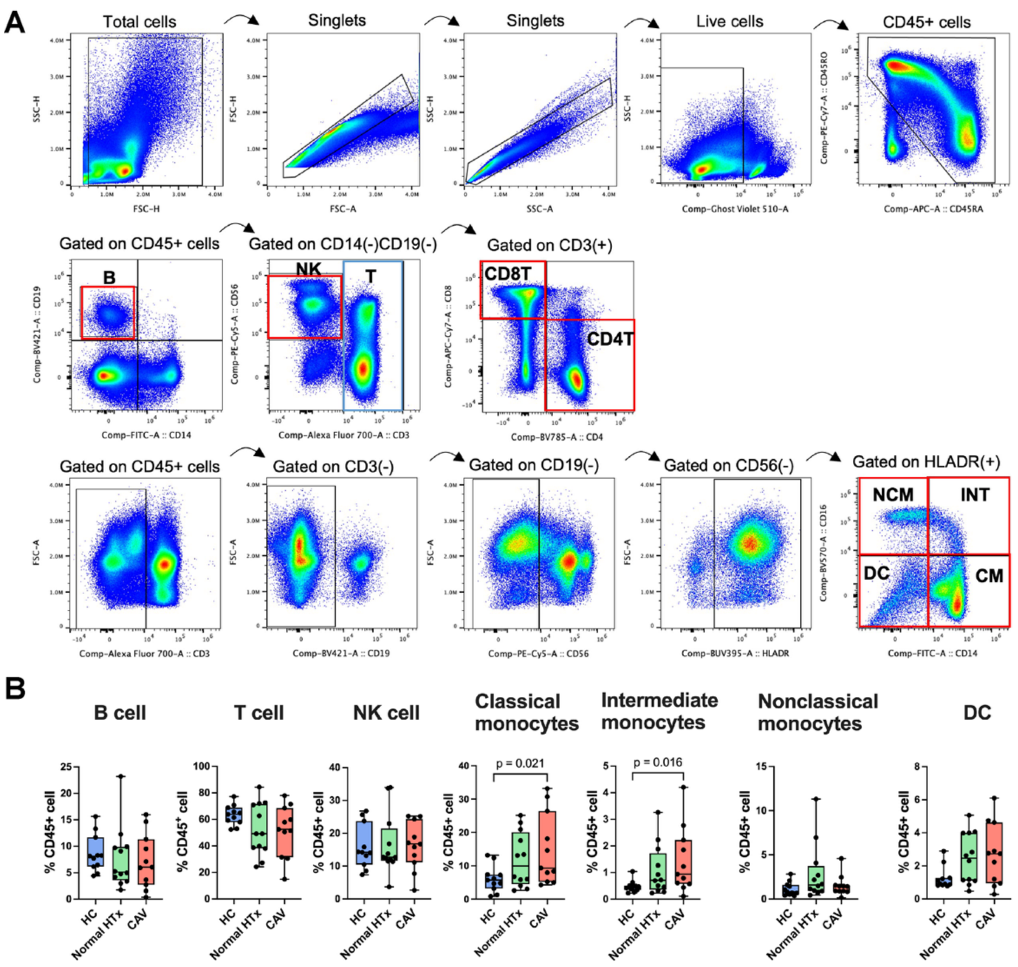

Figure S11. Comparison of major immune cell types by flow cytometry.

A. Plots show the gating of flow cytometry data to obtain major immune cell type populations from PBMCs. Total CD45+ leukocytes were determined in the following gates: non-debris total cells, singlets (FSC-A vs FSC-H, and SSC-A vs SSC-H), viable cells, and CD45+ (CD45RO+ or CD45RA+) cells. CD45+ cells were separated into each major cell type using the gates below.

B: CD14(-)CD19(+)

Natural killer (NK): CD14(-)CD19(-)CD3(-)CD56(+)

CD4$^+$ T: CD14(-)CD19(-)CD3(+)CD8(-)CD4(+)

CD8$^+$ T: CD14(-)CD19(-)CD3(+)CD4(-)CD8(+)

Dendritic cells (DC) and monocytes: CD3(-)CD19(-)CD56(-)HLA-DR(+)

DC: CD14(-)CD16(-)

Classical monocytes: CD14(+)CD16(-),

Intermediate monocytes: CD14(+)CD16(+)

Nonclassical monocytes: CD14(-)CD16(+)

B. The box plots compare the proportions of the major cell types obtained by flow cytometry among HC, normal HTx, and high-grade CAV groups (Dunn's test, unadjusted).

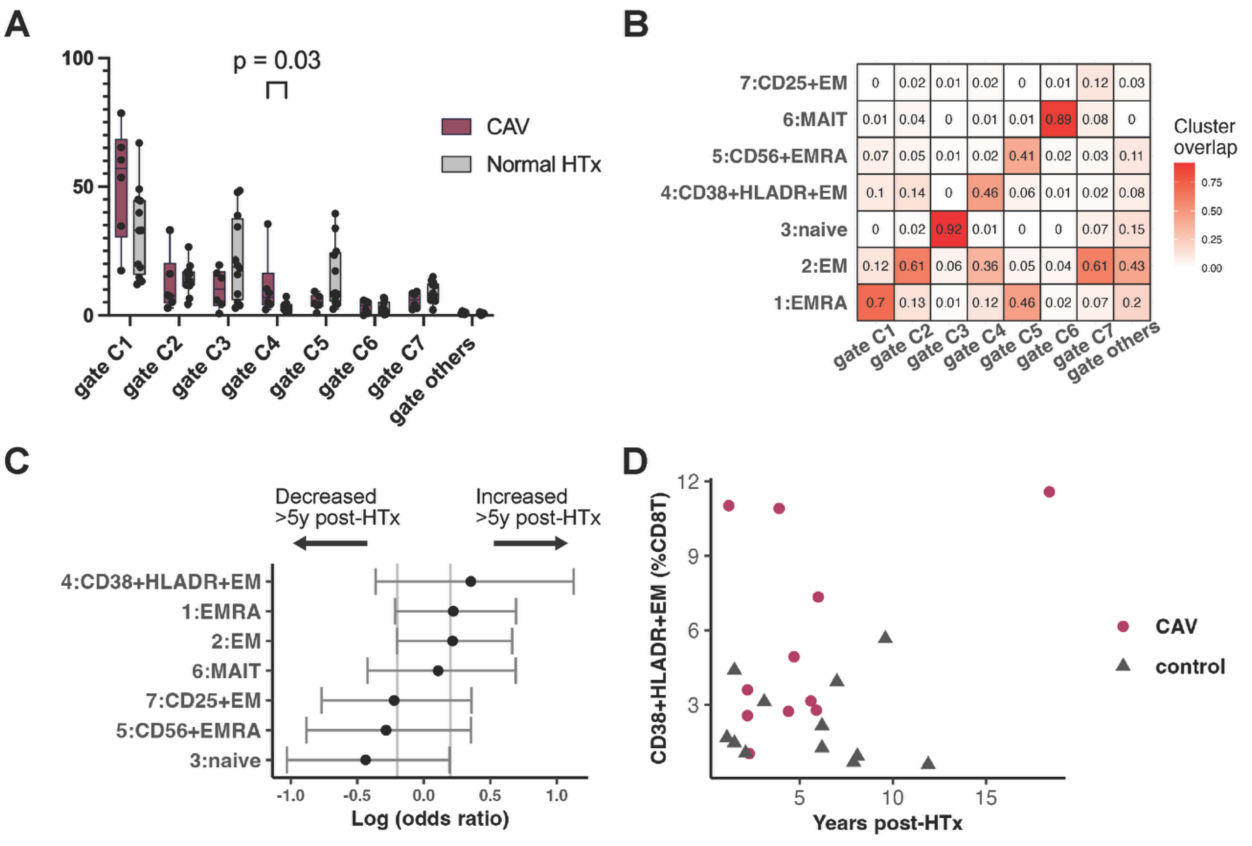

Figure S12. Comparison of the CITE-seq subclusters obtained by gating vs. unsupervised WNN subclusters

A. Proportions of subclusters obtained by applying the gating to the CITE-seq data were compared between high-grade CAV and normal HTx groups. Cells in the CD38$^+$HLA-DR$^+$ CD8$^+$ Tem subcluster determined by the gating (gate C4) were also significantly increased in CAV compared to normal HTx patients (Wilcoxon rank-sum test, unadjusted). **B.** Overlap of cell numbers of each subcluster determined by WNN (rows) and gating (columns) are shown in the heatmap. **C.** The CD38$^+$HLA-DR$^+$ CD8$^+$ Tem subcluster showed no significant difference when comparing samples before versus after 5 years post-HTx from CITE-seq data (log odds ratio estimates with 95% confidence intervals shown). **D.** For flow cytometry, no correlation was seen between proportions of CD38$^+$HLA-DR$^+$ CD8$^+$ Tem cells and years post-HTx (Spearman correlation, p=0.792, rho=-0.06).

A

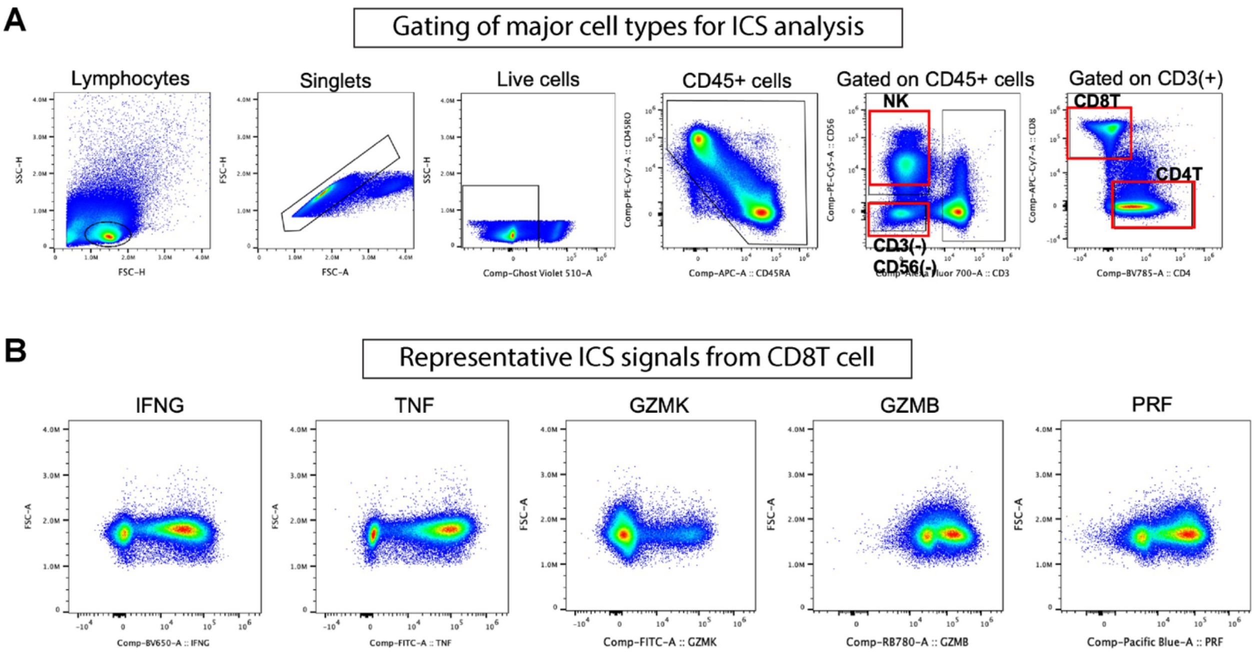

Figure S13. Gating of intracellular cytokine staining for flow cytometry data.

A. Plots show the gating of PBMCs analyzed for the intracellular staining. Lymphocytes were gated and contamination of doublets or non-CD45+ cells were removed by the gating as shown. CD45+ cells were separated by CD3 and CD56 expressions into T cells (CD3+), NK cells (CD3-CD56+), and non-T non-NK cells (CD3-CD56-). From T cells, $CD8^+$ T cells (CD4-CD8+) and $CD4^+$ T cells (CD4+CD8-) were gated. **B.** Representative plots show the expressions of IFNG, TNF, GZMK, GZMB, and PRF in the total $CD8^+$ T cells from a high-grade CAV patient.

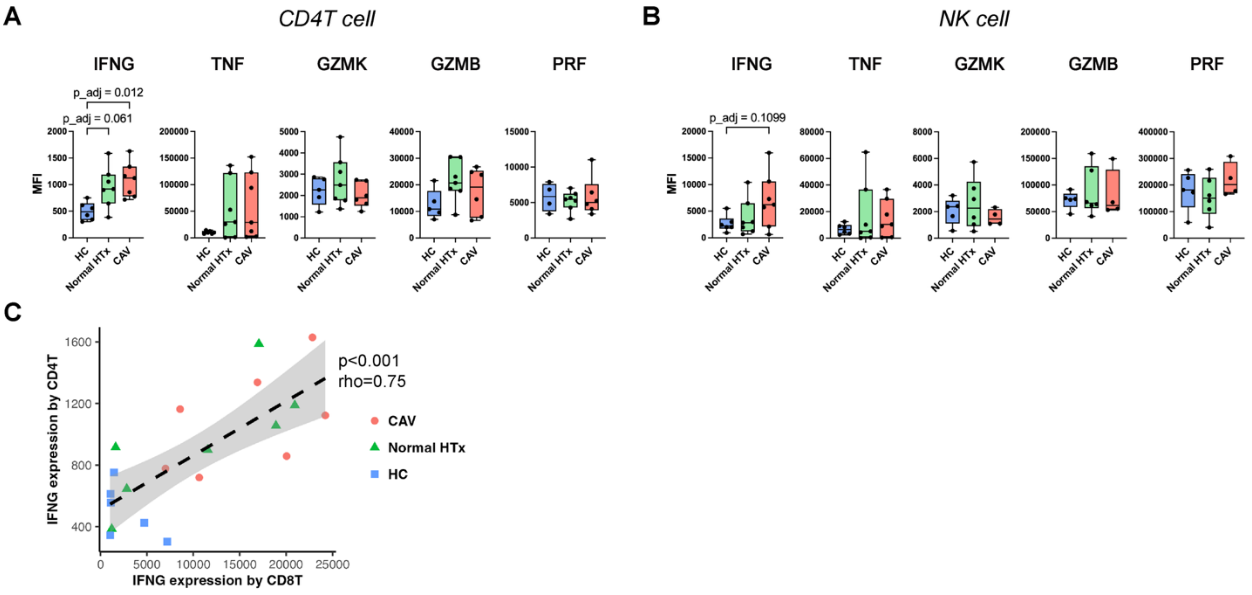

Figure S14. Intracellular cytokine staining for CD4$^+$ T and NK cells.

A and **B.** Box plots compare the expression of the inflammatory and cytotoxic markers in CD4$^+$ T cells (**A**) and NK cells (**B**) among the HC, normal HTx, and high-grade CAV groups. CD4$^+$ T cells from high-grade CAV patients showed significantly higher IFNG expression compared to HC. **C.** The scatter plot shows a significant and strong positive correlation of IFNG expression between CD8$^+$ and CD4$^+$ T cells (Spearman correlation). IFNG and TNF: HC (n=6), normal HTx (n=7), and high-grade CAV (n=7); GZMK, GZMB, and PRF: HC (n=5), normal HTx (n=7), and high-grade CAV (n=6). Pairwise comparison was performed using the Dunn's test for **A** and **B** (p-values adjusted by Bonferroni correction).

Supplementary Excel File 1. The targeted mRNA panel used for CITE-seq

Supplementary Excel File 2. Cell counts for major cell types and $CD8^+$ T, $CD4^+$ T, B, and NK cell subclusters per patient.

Supplementary Excel File 3. Differentially expressed genes across major cell types and $CD8^+$ T, $CD4^+$ T, B, and NK cell subclusters.

Supplementary Excel File 4. Flow cytometry measurements.
